# Multi-omics dataset to decipher the complexity of drug resistance in diffuse large B-cell lymphoma

**DOI:** 10.1038/s41598-018-37273-4

**Published:** 2019-01-29

**Authors:** Luc-Matthieu Fornecker, Leslie Muller, Frédéric Bertrand, Nicodème Paul, Angélique Pichot, Raoul Herbrecht, Marie-Pierre Chenard, Laurent Mauvieux, Laurent Vallat, Seiamak Bahram, Sarah Cianférani, Raphaël Carapito, Christine Carapito

**Affiliations:** 1 Pôle d’Oncologie et d’Hématologie, Hôpitaux Universitaires de Strasbourg, Strasbourg, France; 2 Laboratoire de Spectrométrie de Masse BioOrganique (LSMBO), IPHC, Université de Strasbourg, CNRS UMR 7178 Strasbourg, France; 3 Université de Strasbourg, INSERM, UMR_S1113/IRFAC, Strasbourg, France; 4 Fédération de Médecine Translationnelle de Strasbourg (FMTS), Strasbourg, France; 5 Institut de Recherche Mathématique Avancée, CNRS UMR 7501, LabEx Institut de Recherche en Mathématiques, ses Interactions et Applications, Université de Strasbourg, Strasbourg, France; 6 Laboratoire d’ImmunoRhumatologie Moléculaire INSERM UMR_S1109, Plateforme GENOMAX, Faculté de Médecine, Strasbourg, France; 7 Fédération Hospitalo-Universitaire OMICARE, Université de Strasbourg, Strasbourg, France; 8 Département de Pathologie, Hôpitaux Universitaires de Strasbourg, Strasbourg, France; 9 Laboratoire d’Hématologie, Hôpitaux Universitaires de Strasbourg, Strasbourg, France

## Abstract

The prognosis of patients with relapsed/refractory (R/R) diffuse large B-cell lymphoma (DLBCL) remains unsatisfactory and, despite major advances in genomic studies, the biological mechanisms underlying chemoresistance are still poorly understood. We conducted for the first time a large-scale differential multi-omics investigation on DLBCL patient’s samples in order to identify new biomarkers that could early identify patients at risk of R/R disease and to identify new targets that could determine chemorefractoriness. We compared a well-characterized cohort of R/R versus chemosensitive DLBCL patients by combining label-free quantitative proteomics and targeted RNA sequencing performed on the same tissues samples. The cross-section of both data levels allowed extracting a sub-list of 22 transcripts/proteins pairs whose expression levels significantly differed between the two groups of patients. In particular, we identified significant targets related to tumor metabolism (Hexokinase 3), microenvironment (IDO1, CXCL13), cancer cells proliferation, migration and invasion (S100 proteins) or BCR signaling pathway (CD79B). Overall, this study revealed several extremely promising biomarker candidates related to DLBCL chemorefractoriness and highlighted some new potential therapeutic drug targets. The complete datasets have been made publically available and should constitute a valuable resource for the future research.

## Introduction

Diffuse large B-cell lymphoma (DLBCL) is the most frequent subtype of non-Hodgkin lymphoma (NHL) and is a clinically and biologically heterogeneous disease. The anthracycline-based regimen R-CHOP (rituximab, cyclophosphamide, doxorubicine, vincristine and prednisone) is still considered as the standard of care for first-line treatment with approximately 60% of the patients achieving a complete response. The prognosis of patients with primary refractory or early-relapsed (R/R) disease is particularly poor with a median overall survival below one year. Because of the acquisition of chemoresistance, only a fraction of R/R patients can be cured with salvage therapies^1^.

Recent advances in molecular biology, genetics and high throughput –omics technologies have led to a better understanding of the biology of this disease and the distinction of several subtypes of DLBCL^2^. Based on the cell-of-origin classification, the two major molecular subgroups are germinal center B-cell-like (GCB) and activated B-cell-like (ABC) DLBCL that notably differ in their clinical outcomes^3^. Cytogenetic studies have highlighted the major importance of *MYC, BCL2* and *BCL6* rearrangements^4^. In parallel, the mutational landscape of DLBCL has been extensively studied, demonstrating the intratumoral heterogeneity and allowing the identification of recurrent somatic mutations, some of which provide promising opportunities for new drug developments^5^. However, the mechanisms underlying the resistance to treatment still remain poorly understood and robust biomarkers for the early identification of patients at risk of R/R disease are still lacking.

Mass spectrometry-based proteomics has benefited from an instrumental and methodological revolution over the last two decades. Today, global label-free quantitative proteomic studies enable the identification and quantification of thousands of proteins and provide new opportunities for an in-depth characterization of complex proteomes^6^. As a complement to the static picture revealed by genome sequencing, the comprehensive analysis of the proteome that is dynamic provides crucial information on protein expression to decipher complex biological processes. To date, no data are available in the literature focusing on the proteomic characterization of R/R DLBCL.

In this context, we conducted a large-scale differential proteomic investigation of R/R versus chemosensitive DLBCL patients in order to identify new potential biomarkers related to resistance to treatment and to better understand the biological mechanisms underlying chemoresistance. This proteomic investigation was combined with a quantitative transcriptomics experiment performed on the same samples to correlate genes expression and their impact at the proteomic level.

## Results and Discussion

We performed for the first time a large-scale differential multi-omics study on DLBCL patient’s samples in order to search for new potential biomarkers that could help to early identify patients at risk of R/R disease and to better understand the biological mechanisms underlying chemorefractoriness. In the context of our current knowledge from the literature, a detailed study of some promising new biomarkers is provided below, demonstrating the high value of the present proteogenomic dataset.

Fresh-frozen tumour tissues were collected at the time of diagnosis, before any treatment, for 8 chemorefractory and 12 chemosensitive DLBCL patients who were uniformly treated in first-line with rituximab and an anthracycline-based chemotherapy regimen in a single institution. Patients were considered as chemorefractory if they had a stable or progressive disease after first-line (n = 6), or if they relapsed less than one year after having achieved a complete response (n = 2). Patients who achieved a complete response and did not relapse thereafter, with a minimal follow-up of at least 24 months after the end of treatment, were considered as chemosensitive. Chemorefractory patients were most likely to have an aggressive disease according to the age-adjusted International Prognostic Index (aaIPI) with 87% aaIPI 2–3 in the chemorefractory group and 42% in the chemosensitive group but the difference was not significant (p = 0.07). The two groups did not differ significantly regarding age (p = 0.58), sex (p = 0.64) and Ann Arbor stage (p = 0.16) (Table 1). RNA could be extracted from the same tissue samples that were used for proteomics analysis for 17 patients (7 chemorefractory and 10 chemosensitive). In both groups, the majority of patients were classified into Germinal Center B-Cell-like (GCB) molecular subtype (72% of the chemorefractory patients and 70% of the chemosensitive patients) as determined by rapid reverse transcriptase multiplex ligation-dependent probe amplification assay (RT-MLPA)^7^. The mean percentage of tumor-cells, determined by morphological examination and immunohistochemistry, was 76%, and was ≥70% in 18/20 samples. A single case had a low percentage of tumor cells (20%) but this sample corresponded to a particular subtype of DLBCL (T-cell/histiocyte-rich large B-cell lymphoma). Patient’s characteristics are summarized in Table 1 and a detailed description of the 20 patients is provided in Supplementary Table 1.

**Table 1:** Patients’ characteristics.

	Chemosensitive	Chemorefractory	p value
Age (years)			0.58*
Median	55	57	
range	18–79	31–73	
Sex, n (%)			0.64**
Male	9/12 (75)	5/8 (63)	
Female	3/12 (25)	3/8 (37)	
Ann Arbor Stage, n (%)			0.16**
1–2	6/12 (50)	1/8 (13)	
3–4	6/12 (50)	7/8 (87)	
aaIPI, n (%)			0.07**
0–1	7/12 (58)	1/8 (13)	
2–3	5/12 (42)	7/8 (87)	
Response to first-line, n (%)			<0.01**
Complete response	12/12 (100)	2/8 (25)	
Primary refractory	0/12	6/8 (75)	
Number of treatment lines			<0.01***
Median	1	5	
Range	1–1	3–7	
Cell of origin, n (%)			0.99**
GC	7/10 (70)	5/7 (72)	
ABC	1/10 (10)	1/7 (14)	
Unclassifiable	2/10 (20)	1/7 (14)	

Abbreviations: aaPIP, age-adjusted International Prognostic Index; GC, Germinal Center B-Cell-Like; ABC, Activated B-Cell-Like. *Student’s t-test; **Fisher’s exact test; ***Mann-Whitney test.

Overall, the combined proteomics analysis of the 20 samples resulted in the identification of 4774 unique protein groups (proteins which cannot be unambiguously identified by unique peptides are grouped in one protein group and quantified together). Pairwise comparisons of all samples against each other resulted in a high Pearson coefficient correlation (average r = 0.89) demonstrating a high quantitative accuracy and a high similarity in the global proteomes. The statistical analysis with the peptide-level Robust Ridge Regression model (MSqRob) allowed the relative quantification of 3101 proteins between the two groups of patients, with 586/3101 (18.9%) being significantly differentially abundant with a false discovery rate <5%. Among these differentially abundant proteins, 246 were overexpressed in chemorefractory patients and 340 overexpressed in chemosensitive patients (Supplementary Table 2).

Transcriptomic analysis was performed on 17 samples (these 17 samples are indicated in Supplementary Table 1). It allowed the quantification of 17695 transcripts across the 17 samples. For the 4774 previously identified proteins, the transcript counterpart was also identified by RNAseq in 4338/4774 (90.8%). With an adjusted p-value < 0.1, 244 transcripts were differentially abundant between the two groups of patients (Supplementary Table 3).

Among the 3101 quantified proteins with MSqRob, 2965/3101 (95.6%) were also quantified at the transcriptomic level. The combination of transcriptomics and proteomics data thus resulted in 2965 transcripts/proteins commonly quantified at both levels. When focusing on the 246 proteins overexpressed in chemorefractory patients, only 7 were not identified at the transcriptomic level (Supplementary Table 4) and 24 had a high fold-change (FC) at the proteomic level (log_2_FC (R vs S) >1) and low at the transcriptomic level (log_2_FC (R vs S) <1) (Supplementary Table 5). Conversely, 16 proteins had a low fold-change at the proteomic level (log_2_FC (R vs S) <1) and high at the transcriptomic level (log_2_FC (R vs S) >1) (Supplementary Table 5). When focusing on the 340 proteins overexpressed in the chemosensitive patients, only 11 were not identified at the transcriptomic level (Supplementary Table 4) and 23 had a high fold-change at the proteomic level (log_2_FC (R vs S) <−1) and low at the transcriptomic level (log_2_FC (R vs S) >−1) (Supplementary Table 5). Conversely, only 5 proteins had a low fold-change at the proteomic level (log_2_FC (R vs S) >−1) and high at the transcriptomic level (log_2_FC (R vs S) <−1) (Supplementary Table 5).

By considering only the significantly differentially expressed proteins between the two groups of patients, only 22/586 (3.8%) were found to be also differentially expressed at the transcriptomic level. In all but one case, the variation direction was similar between the two methods with 16/22 transcripts and proteins overexpressed in chemorefractory patients (log_2_FC (R vs S) >0), and 5/22 transcripts and proteins overexpressed in chemosensitive patients (log_2_FC (R vs S) <0) (Table 2). Only one discordant case was observed with Complement C3 that was overexpressed in chemorefractory patient at the proteomics level but overexpressed in chemosensitive patients at the transcriptomics level. Among these 22 differentially expressed transcripts/proteins, 6 were selected and discussed below. The selection was based on a proteomic fold-change threshold (FC (R vs S) >1.5 or <−1.5) and a literature-based strong biological relevance in the context of treatment-resistance in DLBCL.

**Table 2:** Sub-list of 22 differentially abundant transcripts/proteins pairs at both transcriptomics and proteomics levels.

Protein name	Gene name	Proteomics				Transcriptomics			
		Number of patients (n = 20)		log_2_FC (R vs S)	q-value	Number of patients (n = 17)		log_2_FC (R vs S)	adjusted p-value
		R (n = 8)	S (n = 12)			R (n = 7)	S (n = 10)		
C-X-C motif chemokine 13	CXCL13	3	2	2,9	0.006	7	10	1,6	0.019
Indoleamine 2,3-dioxygenase 1	IDO1	6	12	2,9	<0.001	7	10	1,7	0.061
Granzyme H	GZMH	3	2	2,7	0.028	7	10	1,7	0.047
Protein THEMIS2	THEMIS2	7	9	1,2	0.015	7	10	1,3	0.067
Granzyme K	GZMK	8	11	0,6	0.017	7	10	1,5	0.089
Complement C3	C3	8	12	0,3	<0.001	7	10	−1,4	0.060
Hexokinase-3	HK3	8	12	1,7	<0.001	7	10	1,9	0.028
Superoxide dismutase [Mn], mitochondrial	SOD2	8	12	1,2	<0.001	7	10	1,6	0.039
Protein S100-A8	S100A8	8	12	1,7	<0.001	6	9	1,7	0.066
Protein S100-A4	S100A4	8	12	0,9	0.003	7	10	1,4	0.020
PRA1 family protein 3	ARL6IP5	8	12	0,5	0.009	7	10	0,9	0.089
rRNA 2′-O-methyltransferase fibrillarin	FBL	8	12	−0,4	0.006	7	10	−0,8	0.070
40 S ribosomal protein S18	RPS18	8	12	−0,3	0.011	7	10	−1,1	0.076
40 S ribosomal protein S6	RPS6	8	12	−0,3	0.043	7	10	−0,9	0.033
40 S ribosomal protein S12	RPS12	8	12	−0,4	0.009	7	10	−0,9	0.089
Alpha-1-antitrypsin	SERPINA1	8	12	1,1	0.007	7	9	1,8	0.039
Serpin B6	SERPINB6	8	12	1,2	<0.001	7	10	1,5	0.001
Phosphatidylinositol 3,4,5-trisphosphate-dependent Rac exchanger 1 protein	PREX1	8	12	0,8	0.006	7	10	1,1	0.067
Ceruloplasmin	CP	8	12	0,6	<0.001	7	8	1,7	0.083
CD97 antigen	CD97	8	12	0,5	0.003	7	10	1,2	0.074
B-cell antigen receptor complex-associated protein beta chain	CD79B	5	10	−1,7	0.013	7	10	−1,3	0.024
Syntaxin-11	STX11	6	11	1,4	<0.001	7	10	1,2	0.070

Abbreviations: Log_2_FC, log_2_ fold-change; R, chemorefractory patients; S, chemosensitive patients.

**Figure 1:**
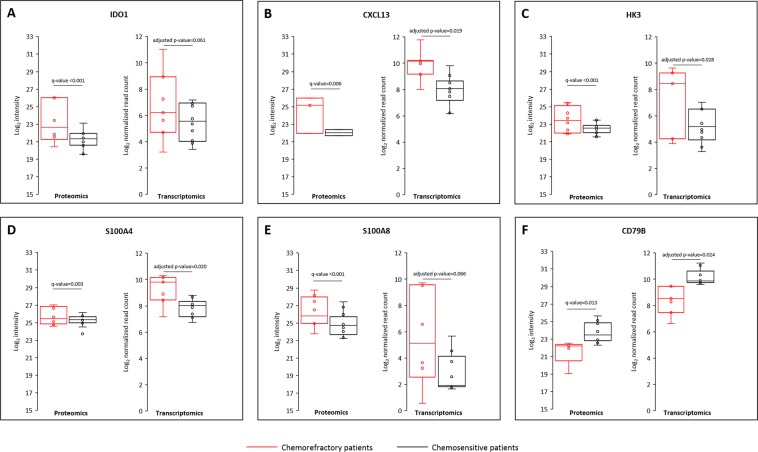
Top six differentially abundant proteins and genes between chemorefractory and chemosensitive patients. For each protein, dots represent the mean of the log_2_ intensities of all the peptides quantified for each patient in each group. For each gene, dots represent the log_2_ normalized read count distribution of the considered gene for each patient in each group.

Indoleamine 2,3-dioxygenase 1 (IDO1) was overexpressed in chemorefractory patients (Fig. 1A). This enzyme is involved in the degradation of the amino acid tryptophan. L-kynurenine, one of the metabolites resulting from tryptophan degradation, has the ability to inhibit T-cell proliferation and to induce T-cell death, contributing to an immunosuppressive microenvironment^8^. Expression of IDO1 evaluated by immunohistochemistry was already found to be positive in one third of DLBCL cases and was associated with a worse response rate and a worse 3-year overall survival after first-line therapy with R-CHOP^9^. From a therapeutic point of view, IDO1 represents a novel immune checkpoint target. Several IDO1 inhibitors (epacadostat (INCB024360), indoximod, navoximod (GDC-0919) or BMS-986205) are now available but failed to demonstrate a therapeutic efficacy as a monotherapy. However, several ongoing trials in various solid tumors (ovarian cancer, pancreatic cancer, squamous cell carcinoma of the head and neck, non-small cell lung cancer, metastatic renal-cell carcinoma for example) are currently evaluating IDO1 inhibitors in combination with other agents such as PD1 or PD-L1 inhibitors^10,11^. Although results from phase 2 studies were encouraging, such as with the combination of epacadostat and anti-PD1 in melanoma patients^12^, recent results from phase 3 studies failed to confirm these results^13^, suggesting that further analysis are warranted to better define the subset of patients who are most likely to benefit from IDO1 inhibitors. No clinical trial is currently ongoing in order to evaluate the potential of IDO1 inhibitors in the context of R/R DLBCL.

We also demonstrated an overexpression of the chemokine C-X-C motif ligand 13 (CXCL13) in chemorefractory patients (Fig. 1B). CXCL13, the unique ligand of CXCR5, is an inflammatory chemokine that contributes to generate a pro-inflammatory microenvironment in angioimmunoblastic T-cell lymphoma^14^. Moreover, it has been shown to be an adverse prognosis factor in advanced colon cancer^15^. In colon cancer cells, the CXCL13-CXCR5 axis participates in tumour growth and invasiveness by activation of the PI3K/AKT signalling pathway^16^. Our results indicate that CXCL13 could also play a key role in the microenvironment of DLBCL.

Hexokinase 3 (HK3) is one of the four isoforms of hexokinase involved in the first step of the glycolysis pathway, converting glucose into glucose-6-phosphate. Glucose metabolism of cancer cells highly differs from that of normal cells. In cancer cells, pyruvate generated by glycolysis is converted into lactate via a phenomenon called “aerobic glycolysis” (Warburg effect)^17^. Overexpression of hexokinase is crucial for cancer cells to produce enough ATP by aerobic glycolysis. Recently, hexokinase 2 was shown to be overexpressed in rituximab-resistant cell lines and to be associated with inhibition of mitochondrial-mediated apoptosis^18^. In our study, hexokinase 2 was identified with a high degree of confidence (by 49 unique peptides covering almost 50% of its sequence) while its expression was not affected between the two groups of patients both at proteomic (log_2_FC = 0.4, q-value = 0.115) and transcriptomic (log_2_FC = 0.7, adjusted p-value = 0.58) levels. Interestingly, hexokinase 3 was also identified with a high degree of confidence (by 29 unique peptides covering 57% of its sequence), and its expression was significantly higher in chemorefractory patients at both proteomics (log_2_FC = 1.7, q-value < 0.001) and transcriptomics levels (log_2_FC = 1.9, adjusted p-value = 0.028) (Fig. 1C). These results suggest that hexokinase 3 could play a key role in DLBCL chemorefractoriness.

Proteins S100 are involved in the regulation of proliferation, migration and invasion of cancer cells, and their dysregulation has been demonstrated in the majority of human cancers^19^. Fifteen S100 family members were identified in our study by proteomic analysis (S100A2, S100A4, S100A6, S100A7, S100A7A, S100A8, S100A9, S100A10, S100A11, S100A12, S100A13, S100A14, S100A16, S100B and S100P) and 4 were found to be significantly overexpressed in chemorefractory patients: S100A4 (log_2_FC = 0.9, q-value = 0.003), S100A8 (log_2_FC = 1.7, q-value < 0.001), S100A9 (log_2_FC = 1.9, q-value < 0.001) and S100A11 (log_2_FC = 0.5, q-value = 0.007). Only S100A4 and S100A8 were significantly overexpressed at both transcriptomics and proteomics levels (Fig. 1D,E). S100A4 and S100A8 were already extensively studied in solid tumours and were found to be associated with tumour growth and metastasis^20,21^. However, few data are available in lymphoma with only S100A9 being described as associated with tumour growth and immune evasion^22^. Our data thus suggest that these proteins could be associated in DLBCL with a more aggressive disease and could participate in the development of resistance to treatment. Based on our results, targeting S100 proteins may represent a therapeutic potential for the treatment of R/R DLBCL.

Finally, the B-cell antigen receptor complex-associated protein beta chain (CD79B) was significantly under-expressed in chemorefractory patients both at proteomic (log_2_FC = −1.7, q-value = 0.013) and transcriptomic (log_2_FC = −1.3, adjusted p-value = 0.023) levels (Fig. 1F). CD79B is necessary for the function of the B-cell receptor and somatic genetic alterations in the *CD79B* gene participate in the constitutive activation of the NF-kB pathway, in particular for the ABC DLBCL subtypes. *CD79B* mutations have been reported in 23% of R/R ABC DLBCL^23^. However, few data are available which investigate the level of CD79B expression. This protein expression level could eventually affect the efficacy of the anti-CD79B antibody-drug conjugate (polatuzumab vedotin) that has recently emerged as a potential active drug in R/R DLBCL^24^.

We used the gene ontology (GO) (http://www.geneontology.org/) and PANTHER database^25^ to perform enrichment analysis based on the Gene Ontology – Biological Process (GO-BP) annotations in our proteomics dataset. This enrichment analysis indicated that proteins significantly overexpressed in chemorefractory patients were particularly enriched in GO-BP associated with inflammation and immune response, as well as the coagulation cascade. At the opposite, proteins significantly overexpressed in chemosensitive patients were enriched in GO-BP associated with ribosome biogenesis and ribosomal RNA (rRNA) processing (Table 3).

**Table 3:** Gene Ontology-Biological Process enrichment among the differentially expressed proteins using the PANTHER database system.

Gene Ontology-Biological Process	Fold enrichment	p value	Fisher’s Exact with FDR multiple test correction
Chemorefractory patients			
Fibrinolysis	8.5	0.0001	<0.0001
Regulation of complement activation	4.1	<0.0001	0.0065
Platelet activation	3.5	0.0002	0.0198
Platelet degranulation	3.2	0.0001	0.0154
Inflammatory response	3.2	<0.0001	0.0001
humoral immune response	3.1	<0.0001	0.0051
Neutrophil degranulation	2.5	<0.0001	<0.0001
negative regulation of immune system process	2.5	0.0004	0.0034
activation of immune response	2.4	<0.0001	0.0018
Chemosensitive patients			
Ribosome biogenesis	3.2	<0.0001	<0.0001
rRNA processing	3.4	<0.0001	<0.0001

Abbreviations: FDR, False Discovery Rate.

These results suggest that cancer-related inflammation and disturbed immune response may play an important role and contribute to chemorefractoriness in DLBCL. The role of cancer-related inflammation in the development and progression of tumors, as well as in patient outcomes, has been recognized since many years^26^. In addition to CXCL13 and S100A8 proteins, that were previously discussed, the other proteins from our dataset that were found to be significantly overexpressed at proteomics and transcriptomics level in chemorefractory patients and involved in inflammation or immune response are Granzyme H, Granzyme K, Complement C3 and Alpha-1-antitrypsin. Our results also suggest that a local activation of coagulation may contribute to treatment-resistance and tumor progression, and are consistent with previously published works having already highlighted the role of blood coagulation in cancer progression. In particular, it has been shown that blood coagulation enzymes in the tumor microenvironment played a role in solid-tumor progression and metastasis^27,28^. These results highlight the potential major role played by the microenvironment in tumor progression and drug-resistance in DLBCL. The strong interactions between cancer-cells and their surrounding microenvironment have been already largely studied, and targeting the microenvironment offers now novel therapeutic perspectives in cancer^29,30^. In this study, proteomic and transcriptomic analysis were performed on whole tissue sections, thus allowing the analysis of DLBCL-cells and their microenvironment. One major drawback of this approach is that these two compartments cannot be analysed separately, but this type of approach could be considered as an opportunity, in the context of a biomarker discovery study, to provide the most exhaustive list of potential new biomarkers. However, in this context, validation studies are a crucial need in order to determine more precisely the relative contribution of both compartments that are tumor-cells and microenvironment. To achieve such an objective, immunohistochemistry could be considered as a method of choice.

In this study, we present the first high-throughput multi-omics study in DLBCL. Over the past two decades, genomics and transcriptomics have largely dominated in cancer research, in particular with the advent of next-generation sequencing (NGS) technologies. In 2000, gene-expression profiling allowed the clear distinction of two molecular DLBCL subtypes, namely Activated B-Cell (ABC) and Germinal Center B cell-Like (GCB) subtypes^3^. The development of NGS technologies resulted in the recent publication of exome sequencing in 1001 DLBCL patients allowing to depict the nearly complete mutational landscape in DLBCL and the identification of 150 driver genes^31^. This in-depth and extensive molecular characterization of DLBCL at genomic and transcriptomic levels also recently led to the proposal of novel molecular classifications in DLBCL identifying subgroups of patients with distinct clinical behaviour and prognosis^32,33^. More recently, MS-based proteomics has emerged as an important tool for the characterization of DLBCL. Various methodological approaches have been used with an aim to exploring various aspects of the disease, such as pathogenesis, subtypes classification or therapeutic issues. Super-SILAC-based approaches have demonstrated the ability to distinguish DLBCL subtypes according to their cell of origin in patient-derived DLBCL cell lines as well as on tumor samples from patients^34–36^. So far, few proteomics studies have addressed the drug-resistance challenge in DLBCL. This issue has been first addressed by a proteomic study (two-dimensional gel electrophoresis with MALDI-TOF/TOF-MS analysis) aiming to identify differential proteins expressed by DLBCL cells with high or low sensitivity to chemotherapy after *in vitro* exposure to the CHOP regimen compounds. Nineteen differentially expressed proteins were identified between the two groups. Among these differentially expressed proteins, immunohistochemical analysis performed in DLBCL tissue samples from 98 patients confirmed a higher expression of Glutathione S-transferase (GSTP1) and Heat shock protein beta-1 (HSPB1), and a lower expression of Ezrin (EZR) and Pleckstrin (PLEK) in patients with relapse or progressive disease after CHOP chemotherapy^37^. In another study, by using a SILAC-based quantitative proteomic approach on 10 DLBCL patients selected according to their response to treatment (5 patients with primary refractory disease or early relapse, and 5 patients considered cured), 87 proteins, among a total of 3027 successfully quantified proteins, were differentially expressed between the two groups of patients with 21 overexpressed in refractory patients. The authors could demonstrate an up-regulation of proteins involved in the regulation of the actin cytoskeleton in chemosensitive patients^38^. This work was pursued by using a tandem mass tag (TMT)-based quantitative proteomic approach performed on microdissected samples obtained from formalin-fixed paraffin-embedded tissues. This study allowed identifying 102 DA proteins and the authors could confirm the up-regulation of proteins involved in actin regulation in chemosensitive patients. Interestingly, they managed to highlight a potential role for ribosomal proteins in treatment-resistance as these proteins were largely represented in those found to be overexpressed in chemorefractory patients^39^. In comparison with these previously published works, our study points out the potential role of the microenvironment in drug-resistance in DLBCL. However, we observed one discrepancy between our study and the study published by Bram Ednersson *et al*. regarding the potential role of ribosomal proteins. In our study, ribosomal proteins and ribosome biogenesis appeared over-represented in chemosensitive patients, while it was the opposite in the work of Bram Ednersson *et al*. There is no obvious explanation, but it remains hazardous to make a direct comparison between two exploratory studies that differ in several technical and methodological aspects such as the tissue used for protein extraction (fresh-frozen vs formalin-fixed paraffin-embedded, whole-tissue vs microdissection), quantitative proteomic workflow (label-free vs super-SILAC) and statistical analysis. Nevertheless, these two studies point out a potential role for ribosome proteins in drug-resistance and, therefore, warrant continued research to clarify the role of these proteins in treatment-resistance of DLBCL.

In conclusion, this study revealed several extremely promising biomarker candidates associated with chemorefractoriness, related to tumour metabolism, microenvironment, BCR signalling pathway, hence highlighting new potential therapeutic drug targets. The combination of multilevel –omics datasets is very useful to reduce lists of thousands of candidates to a subset of significant targets, as well as to cross-validate candidates by different techniques. Further studies will be necessary to validate these findings in a larger and independent cohort of patients. However, the present work already provides greater insights in the underlying mechanisms of chemoresistance in DLBCL, supported by a publically available dataset.

## Materials and Methods

### Patients selection

Patients were selected among the fresh-frozen tissue-sample collection available from the “Centre de ressources Biologiques des Hôpitaux Universitaires de Strasbourg”. We retrospectively analyzed the treatment and outcome of each patient. Only patients for whom a tissue-sample collected at the time of diagnosis and treated in first-line with the combination of anti-CD20 monoclonal antibody and an anthracycline-based regimen were selected. Patients were considered as chemorefractory if they had a stable or progressive disease after first-line, or if they relapsed less than one year after having achieved a complete response. Patients who achieved a complete response after first-line and did not relapse thereafter, with a minimal follow-up of at least 24 months after the end of treatment, were considered as chemosensitive.

### Samples handling

All samples were obtained by a surgical resection or radiological-guided biopsy from a tumor mass. Proteomic and transcriptomic analysis were performed on the same specimens that were used for the diagnosis of the disease. After collection, all samples were stored at −80 °C, without conservative medium, until protein or RNA extraction. The neoplastic content in each sample was determined by a pathological review of all cases, based on morphology and immunohistochemistry for distinguishing B-cells and T-Cells.

### Proteomics analysis

#### Sample preparation

Proteins were extracted from ~10 mg of fresh frozen tumor tissues in a lysis buffer containing 62.5 mM Tris HCl pH 6.8, 2% SDS and 10% glycerol. Protein concentration was determined with DC^TM^ method (Bio-Rad) according to manufacturer’s instructions. For each sample, 20 µg of proteins were used for tube-gel preparation, as previously described^40^. Briefly, 7.5% acrylamide/Bis-acrylamide, and 0.25 µL TEMED were added for a final volume of 100 μL. Ammonium persulfate (2.50 µL) was added to initiate polymerization. After fixation with 50% ethanol/3% phosphoric acid, tube-gels were cut in 2 mm sections and each section in ~2 mm^2^ pieces. The gel pieces were washed and the cysteine residues were reduced by adding 10 mM DTT for 30 min at 60 °C and 30 min at room temperature, and alkylated by adding 55 mM IAA for 20 min in the dark. The gel pieces were then washed three times by adding 50/50 (v/v) 25 mM NH_4_HCO_3_/acetonitrile (ACN). After two dehydrations with ACN, the proteins were cleaved in an adequate volume to cover all gel pieces with a modified porcine trypsin (Promega) solution at a 1:80 (w/w) enzyme:protein ratio. Digestion was performed overnight at 37 °C. Tryptic peptides were extracted twice under agitation, first with 60% ACN in 0.1% FA for 1 h and then with 100% ACN for 1 h. The excess of ACN was vacuum dried, and the samples were resolubilized with H_2_O/ACN/FA (98/2/0.1 v/v/v).

#### NanoLC-MS/MS analysis

The nanoLC-MS/MS analysis was performed on a nanoAcquity UPLC device (Waters Corporation, Milford, USA) coupled to a Q-Exactive Plus mass spectrometer (Thermo Fisher Scientific, Waltham, Massachusetts, USA). Peptide separation was performed on an ACQUITY UPLC BEH130 C18 column (250 mm × 75 μm with 1.7 μm diameter particles) and a Symmetry C18 precolumn (20 mm × 180 μm with 5 μm diameter particles, Waters). The solvent system consisted of 0.1% FA in water (solvent A) and 0.1% FA in ACN (solvent B). Samples (equivalent to 800 ng of proteins) were loaded into the enrichment column over 3 min at 5 μL/min with 99% of solvent A and 1% of solvent B. The peptides were eluted at 450 nL/min with the following gradient of solvent B: from 1 to 35% over 120 min and 35 to 80% over 1 min. The 20 samples were injected in randomized order. The MS capillary voltage was set to 1.8 kV at 250 °C. The system was operated in Data Dependent Acquisition mode with automatic switching between MS (mass range 300–1800 m/z with R = 140,000, Automatic gain control (AGC) fixed at 3 × 10^6^ ions and a maximum injection time set at 50 ms) and MS/MS (mass range 200–2000 m/z with R = 17,500, AGC fixed at 1 × 10^5^ and the maximal injection time set to 100 ms) modes. The ten most abundant peptides were selected on each MS spectrum for further isolation and higher energy collision dissociation fragmentation, excluding unassigned and monocharged ions. The dynamic exclusion time was set to 60 s.

#### Data analysis

Raw data obtained for each sample were processed using MaxQuant (version 1.5.5.1). Peaks were assigned with the Andromeda search engine with full trypsin specificity. The isoform-containing human database used for the search was extracted from the UniProtKB-SwissProt database (26 sept 2016, 42,144 entries). The minimum peptide length required was seven amino acids and a maximum of one missed cleavage was allowed. Methionine oxidation was set as a variable modification and peptides with modified methionines, as well as their unmodified counterparts, were excluded from protein quantification. Cysteine carbamidomethylation was set as a variable modification to account for the potential propionamide modifications of cysteine residues. Cysteine propionamidation was thus also set as a variable modification. For protein quantification, the “match between runs” option was enabled. The maximum false discovery rate was 1% at peptide and protein levels with the use of a decoy strategy. We used the “peptides.txt” files exported from MaxQuant for further statistical analysis with the peptide-level Robust Ridge Regression model (MSqRob) R-package^41^.

Statistical analysis was performed for all quantified proteins and transcripts, independently of the number of patients in whom the proteins and/or transcripts were quantified.

The mass spectrometry proteomics data have been deposited in the ProteomeXchange Consortium database with the identifier PXD009089^42^.

### Transcriptomics analysis

#### RNA isolation and sequencing

Total RNA was isolated from fresh frozen tumor tissues with the with TRIzol reagent (Invitrogen, Carlsbad, California, USA). RNA extraction could not be performed for 3 patients (samples #5, #12 and #15 in supplementary Table 1) because of the low quantity of available tissue. RNA integrity was assessed with the Agilent total RNA Pico Kit on a 2100 Bioanalyzer instrument (Agilent Technologies, Paolo Alto, USA). The sequencing library was prepared with the Ion AmpliSeq Transcriptome Human Gene Expression Panel (Thermo Fisher Scientific, Waltham, Massachusetts, USA) according to the manufacturer’s protocol^43^. Briefly, after reverse transcription of total RNA, the cDNAs were amplified by multiplex PCR including a total of 20,812 amplicons. These amplicons were then partially digested, and after barcoded sequencing adapter ligation, the libraries were loaded at a concentration of 75 pM on an Ion PI IC 200 chip using the Ion Chef Instrument (Thermo Fisher Scientific, Waltham, Massachusetts, USA). Finally, the sequencing took place on an Ion Proton sequencer with the Ion PI IC 200 Kit, according to the manufacturer’s instructions (Thermo Fisher Scientific, Waltham, Massachusetts, USA).

#### Analysis of RNA-sequence reads

The raw reads were processed by the Torrent Suite analysis pipeline and mapped to the human genome assembly hg19 AmpliSeqTranscriptome. The Torrent AmpliSeqRNA Plugin was used to generate raw read counts which were further used for differential analysis. An average of 3.5 million reads were generated per sample with 93.25% reads on target and an average of 11,005 amplicons covered by at least 10 reads. We applied the R Bioconductor package DESeq2 to identify genes that were differentially expressed. The gene selection was based on the adjusted p-value. All genes with an adjusted p-value lower than 0.1 were selected as differentially expressed^44^.

RNAseq raw data have been deposited in fastaq format in the EMBL-EBI ArrayExpress archive (https://www.ebi.ac.uk/arrayexpress/) with the accession number E-MTAB-6597.

### Ethical Committee

Sample collection for further research analysis was approved by an Ethical Committee (“Comité de Protection des Personnes Est IV”, Strasbourg, France) and all patients provided an informed consent according to the Declaration of Helsinki.

## Supplementary information

Supplementary Tables
