# Supplementary material for: Multi-omics dataset to decipher the complexity of drug resistance in diffuse large B-cell lymphoma
Source: Sci Rep. 2019 Jan 29;9:895. doi: 10.1038/s41598-018-37273-4 (PMC6351558; doi:10.1038/s41598-018-37273-4)
Supplement: Supplementary file 1 — Supplementary Tables [file 41598_2018_37273_MOESM1_ESM.pdf]

Multi-omics dataset to decipher the complexity of drug resistance in diffuse large B-cell lymphoma

Luc-Matthieu Fornecker,[1-4*] Leslie Muller,[2*] Frédéric Bertrand,[5] Nicodème Paul,[4,6,7] Angélique Pichot,[4,6,7] Raoul Herbrecht,[1,3,4] Marie-Pierre Chenard,[4,8] Laurent Mauvieux,[3,4,9] Laurent Vallat,[3,4,9] Seiamak Bahram,[4,6,7] Sarah Cianférani,[2,7] Raphaël Carapito,[4,6,7] and Christine Carapito[2,7]

[1]Pôle d'Oncologie et d'Hématologie, Hôpitaux Universitaires de Strasbourg, Strasbourg, France. [2]Laboratoire de Spectrométrie de Masse BioOrganique (LSMBO), IPHC, Université de Strasbourg, CNRS UMR 7178, Strasbourg, France. [3]Université de Strasbourg, INSERM, UMR_S1113/IRFAC, Strasbourg, France. [4]Fédération de Médecine Translationnelle de Strasbourg (FMTS), Strasbourg, France. [5]Institut de Recherche Mathématique Avancée, CNRS UMR 7501, LabEx Institut de Recherche en Mathématiques, ses Interactions et Applications, Université de Strasbourg, Strasbourg, France. [6]Laboratoire d'ImmunoRhumatologie Moléculaire INSERM UMR_S1109, Plateforme GENOMAX, Faculté de Médecine, Strasbourg, France. [7]Fédération Hospitalo-Universitaire OMICARE, Université de Strasbourg, Strasbourg, France. [8]Département de Pathologie, Hôpitaux Universitaires de Strasbourg, Strasbourg, France. [9]Laboratoire d'Hématologie, Hôpitaux Universitaires de Strasbourg, Strasbourg, France.

[*]these two authors equally contributed to this work

Supplementary Table 1. Detailed description of the 20 samples.

	Patient	Age (years)	Sex	Histological diagnosis	Molecular sub-type	Tissue sample	% of Tumor cells	aaIPI	Ann Arbor Stage	Transcriptomics analysis	Number of treatment lines
**Chemorefractory**	**#1**	34	M	T-cell/histiocyte-riche DLBCL	GC	LN	20	2	4	Yes	5
	**#2**	50	M	DLBCL NOS	GC	LN	70	2	4	Yes	4
	**#3**	31	M	DLBCL NOS	GC	LN	50	2	4	Yes	3
	**#4**	63	M	DLBCL NOS	ABC	LN	70	3	4	Yes	7
	**#5**	73	F	DLBCL NOS	/	Muscle	70	2	4	No	3
	**#6**	49	F	DLBCL NOS	GC	Mesenteric mass	75	2	4	Yes	5
	**#7**	69	M	DLBCL NOS	NC	LN	90	2	4	Yes	6
	**#8**	67	F	DLBCL NOS	GC	LN	70	1	2	Yes	3
**Chemosensitive**	**#9**	46	M	DLBCL NOS	NC	LN	75	0	1	Yes	1
	**#10**	65	M	DLBCL Burkitt-like	GC	LN	85	0	2	Yes	1
	**#11**	72	M	DLBCL NOS	GC	LN	80	2	4	Yes	1
	**#12**	29	M	DLBCL NOS	/	Bone	85	2	4	No	1
	**#13**	63	F	DLBCL NOS	ABC	LN	80	0	2	Yes	1
	**#14**	56	M	DLBCL NOS	NC	Vallecular masse	80	0	2	Yes	1
	**#15**	32	M	DLBCL NOS	/	Peri-gastric masse	90	1	2	No	1
	**#16**	18	M	Primary mediastinal DLBCL	GC	LN	85	2	4	Yes	1
	**#17**	24	F	Primary mediastinal DLBCL	GC	LN	85	2	4	Yes	1
	**#18**	54	M	DLBCL NOS	GC	Mesenteric mass	90	0	2	Yes	1
	**#19**	59	M	DLBCL Burkitt-like	GC	LN	80	1	3	Yes	1
	**#20**	79	F	DLBCL NOS	GC	LN	80	3	4	Yes	1

Abbreviations: DLBCL, Diffuse Large C-Cell Lymphoma; NOS, Not Otherwise Specified; aaIPI, age-adjusted International Prognostic Index; GC, Germinal Center; ABC, Activated B-Cell; LN, Lymph Node; M, Male; F, female

Supplementary Table 2. Differentially abundant proteins between the two groups of patients

Gene name	Protein goup description	Accession number	log2FC (R vs S)	q-value
A2M	Alpha-2-macroglobulin	P01023	0.3	0.0105
AARSD1	Alanyl-tRNA editing protein Aarsd1	Q9BTE6;Q9BTE6-2;Q9BTE6-3	-0.4	0.0227
AASDHPPT	L-aminoadipate-semialdehyde dehydrogenase-phosphopantetheinyl transferase	Q9NRN7	0.6	0.0259
ABI3BP	Target of Nesh-SH3	Q7Z7G0	-1.1	0.0354
ACAA1	3-ketoacyl-CoA thiolase, peroxisomal	P09110	0.7	0.0046
ACAP1	Arf-GAP with coiled-coil, ANK repeat and PH domain-containing protein 1	Q15027	0.4	0.0102
ACO1	Cytoplasmic aconitate hydratase	P21399	0.6	<0.0001
ADNP	Activity-dependent neuroprotector homeobox protein	Q9H2P0	-0.4	0.0191
ADPRH	[Protein ADP-ribosylarginine] hydrolase	P54922	0.9	0.0323
AHNAK	Neuroblast differentiation-associated protein AHNAK	Q09666	0.1	0.0008
AHSA1	Activator of 90 kDa heat shock protein ATPase homolog 1	O95433	-1.0	0.0404
AK4	Adenylate kinase 4, mitochondrial	P27144	0.7	0.0071
AKR1A1	Alcohol dehydrogenase [NADP(+)]	P14550	0.3	0.0022
AKR1B1	Aldose reductase	P15121	0.4	0.0160
ALDH18A1	Delta-1-pyrroline-5-carboxylate synthase;Gamma-glutamyl phosphate reductase;Glutamate 5-kinase	P54886-2;P54886	-0.3	0.0255
ALDH1B1	Aldehyde dehydrogenase X, mitochondrial	P30837	-0.5	0.0142
ALDOC	Fructose-bisphosphate aldolase C	P09972	0.3	0.0418
ANKRD13A	Ankyrin repeat domain-containing protein 13A	Q8IZ07	-0.5	0.0020
ANKRD22	Ankyrin repeat domain-containing protein 22	Q5VYY1	2.5	0.0014
ANXA1	Annexin A1	P04083	0.3	0.0012
ANXA2	Annexin A2	P07355;P07355-2	0.3	0.0314
ANXA5	Annexin A5	P08758	0.6	<0.0001

ANXA6	Annexin A6	P08133	1.1	0.0144
AOC3	Membrane primary amine oxidase	Q16853;Q16853-2	-0.8	0.0084
APEH	Acylamino-acid-releasing enzyme	P13798	0.2	0.0216
APOA4	Apolipoprotein A-IV	P06727	-0.5	<0.0001
APOB	Apolipoprotein B-100;Apolipoprotein B-48	P04114	0.5	<0.0001
APOBEC3G	DNA dC->dU-editing enzyme APOBEC-3G	Q9HC16	1.1	<0.0001
APOE	Apolipoprotein E	P02649	0.4	0.0089
APOL2	Apolipoprotein L2	Q9BQE5	0.6	0.0157
APOL3	Apolipoprotein L3	O95236-2;O95236	1.3	0.0398
APRT	Adenine phosphoribosyltransferase	P07741	1.0	0.0094
ARHGAP1	Rho GTPase-activating protein 1	Q07960	0.3	0.0086
ARHGAP4	Rho GTPase-activating protein 4	P98171;P98171-2	0.3	0.0004
ARL6IP5	PRA1 family protein 3	O75915	0.5	0.0094
ASPH	Aspartyl/asparaginyl beta-hydroxylase	Q12797-10;Q12797	0.7	0.0240
ATL3	Atlastin-3	Q6DD88	0.4	0.0003
ATP1A1	Sodium/potassium-transporting ATPase subunit alpha-1	P05023-3;P05023-4;P05023;P05023-2	-0.3	0.0087
ATP5H	ATP synthase subunit d, mitochondrial	O75947-2;O75947	-0.6	<0.0001
AZGP1	Zinc-alpha-2-glycoprotein	P25311	0.3	0.0460
BCLAF1	Bcl-2-associated transcription factor 1	Q9NYF8-4;Q9NYF8-3;Q9NYF8-2;Q9NYF8	-0.4	0.0216
BGN	Biglycan	P21810	-1.1	<0.0001
BIN2	Bridging integrator 2	Q9UBW5-2;Q9UBW5	0.5	0.0041
BLK	Tyrosine-protein kinase Blk	P51451	1.1	0.0040
BLVRA	Biliverdin reductase A	P53004	0.5	0.0001
BOP1	Ribosome biogenesis protein BOP1	Q14137-2;Q14137	-0.5	0.0028
BPGM	Bisphosphoglycerate mutase	P07738	-1.0	0.0498
BPI	Bactericidal permeability-increasing protein	P17213	-3.1	0.0006
BPNT1	3'(2'),5'-bisphosphate nucleotidase 1	O95861;O95861-4;O95861-2	-0.8	0.0072

BST2	Bone marrow stromal antigen 2	Q10589-2;Q10589	-0.9	0.0006
BTAF1	TATA-binding protein-associated factor 172	O14981	-0.4	0.0313
BYSL	Bystin	Q13895	-0.5	0.0007
C1QA	Complement C1q subcomponent subunit A	P02745	1.1	0.0328
C1QBP	Complement component 1 Q subcomponent-binding protein, mitochondrial	Q07021	-0.5	0.0441
C3	Acylation stimulating protein;C3a anaphylatoxin;C3-beta-c;Complement C3;Complement C3 alpha chain;Complement C3 beta chain;Complement C3b alpha' chain;Complement C3c alpha' chain fragment 1;Complement C3c alpha' chain fragment 2;Complement C3d fragment;Complement C3dg fragment;Complement C3f fragment;Complement C3g fragment	P01024	0.3	<0.0001
C4A	C4a anaphylatoxin;C4b-A;C4d-A;Complement C4 beta chain;Complement C4 gamma chain;Complement C4-A;Complement C4-A alpha chain	P0C0L4;P0C0L4-2	1.2	0.0209
C4BPA	C4b-binding protein alpha chain	P04003	1.0	0.0001
C5	C5a anaphylatoxin;Complement C5;Complement C5 alpha chain;Complement C5 alpha' chain;Complement C5 beta chain	P01031	0.6	0.0014
C9	Complement component C9;Complement component C9a;Complement component C9b	P02748	0.7	0.0109
C9orf142	Protein PAXX	Q9BUH6	0.4	0.0326
CAD	Aspartate carbamoyltransferase;CAD protein;Dihydroorotase;Glutamine-dependent carbamoyl-phosphate synthase	P27708	-0.2	0.0117
CALD1	Caldesmon	Q05682-5;Q05682-4;Q05682-3;Q05682-6;Q05682-2;Q05682	-0.9	<0.0001

CAMP	Antibacterial protein FALL-39;Antibacterial protein LL-37;Cathelicidin antimicrobial peptide	P49913	-1.6	0.0078
CAPN1	Calpain-1 catalytic subunit	P07384	0.2	0.0014
CAPRIN1	Caprin-1	Q14444-2;Q14444	-0.4	0.0003
CAT	Catalase	P04040	-0.3	0.0047
CBR1	Carbonyl reductase [NADPH] 1	P16152	0.6	0.0161
CCAR1	Cell division cycle and apoptosis regulator protein 1	Q8IX12-2;Q8IX12	-0.3	0.0039
CCDC124	Coiled-coil domain-containing protein 124	Q96CT7	-0.5	0.0022
CCDC58	Coiled-coil domain-containing protein 58	Q4VC31	-0.6	0.0018
CD14	Monocyte differentiation antigen CD14;Monocyte differentiation antigen CD14, membrane-bound form;Monocyte differentiation antigen CD14, urinary form	P08571	0.7	0.0068
CD38	ADP-ribosyl cyclase/cyclic ADP-ribose hydrolase 1	P28907	0.6	0.0120
CD79B	B-cell antigen receptor complex-associated protein beta chain	P40259;P40259-3	-1.7	0.0128
CD8A	T-cell surface glycoprotein CD8 alpha chain	P01732-2;P01732;P01732-3	1.4	0.0002
CD9	CD9 antigen	P21926	-0.9	0.0160
CD97	CD97 antigen;CD97 antigen subunit alpha;CD97 antigen subunit beta	P48960-2;P48960-3;P48960	0.5	0.0033
CDC5L	Cell division cycle 5-like protein	Q99459	-0.3	0.0028
CDK6	Cyclin-dependent kinase 6	Q00534	-0.9	0.0014
CDS2	Phosphatidate cytidylyltransferase 2	O95674	0.7	0.0004
CES1	Liver carboxylesterase 1	P23141-3;P23141;P23141-2	1.3	<0.0001
CFB	Complement factor B;Complement factor B Ba fragment;Complement factor B Bb fragment	P00751	0.6	0.0022
CFH	Complement factor H	P08603	0.5	0.0028
CHD4	Chromodomain-helicase-DNA-binding protein 4	Q14839;Q14839-2	-0.4	<0.0001
CHERP	Calcium homeostasis endoplasmic reticulum protein	Q8IWX8	-0.4	0.0005

CILP	Cartilage intermediate layer protein 1;Cartilage intermediate layer protein 1 C1;Cartilage intermediate layer protein 1 C2	O75339	-1.5	<0.0001
CKAP4	Cytoskeleton-associated protein 4	Q07065	-0.3	0.0041
CLIC1	Chloride intracellular channel protein 1	O00299	0.7	<0.0001
CLIP2	CAP-Gly domain-containing linker protein 2	Q9UDT6-2;Q9UDT6	-1.8	0.0073
CLPX	ATP-dependent Clp protease ATP-binding subunit clpX-like, mitochondrial	O76031	-0.4	0.0301
CLU	Clusterin;Clusterin alpha chain;Clusterin beta chain	P10909-4;P10909;P10909-5;P10909-2	1.2	0.0001
CLUH	Clustered mitochondria protein homolog	O75153	-0.6	0.0064
COL12A1	Collagen alpha-1(XII) chain	Q99715;Q99715-4	-1.7	<0.0001
COL14A1	Collagen alpha-1(XIV) chain	Q05707-2;Q05707	-0.7	0.0493
COL4A1	Arresten;Collagen alpha-1(IV) chain	P02462-2;P02462	1.4	0.0008
COL6A1	Collagen alpha-1(VI) chain	P12109	-0.4	<0.0001
COTL1	Coactosin-like protein	Q14019	0.4	0.0216
CP	Ceruloplasmin	P00450	0.6	<0.0001
CPNE1	Copine-1	Q99829	0.3	0.0278
CPPED1	Serine/threonine-protein phosphatase CPPED1	Q9BRF8	0.7	0.0321
CPSF7	Cleavage and polyadenylation specificity factor subunit 7	Q8N684-2;Q8N684;Q8N684-3	-0.4	0.0022
CPVL	Probable serine carboxypeptidase CPVL	Q9H3G5	0.8	0.0019
CR1	Complement receptor type 1	P17927	0.8	0.0216
CRTAP	Cartilage-associated protein	O75718	-0.6	0.0357
CSDE1	Cold shock domain-containing protein E1	O75534-2;O75534;O75534-3;O75534-4	-0.2	0.0205
CSTA	Cystatin-A;Cystatin-A, N-terminally processed	P01040	1.4	0.0310
CSTF2	Cleavage stimulation factor subunit 2	P33240-2;P33240	-0.4	0.0390
CTSB	Cathepsin B;Cathepsin B heavy chain;Cathepsin B light chain	P07858	0.7	0.0057

CTSZ	Cathepsin Z	Q9UBR2	0.5	0.0090
CUL1	Cullin-1	Q13616	-0.3	0.0002
CUL5	Cullin-5	Q93034	-0.3	0.0474
CXCL13	C-X-C motif chemokine 13	O43927	2.9	0.0055
DCK	Deoxycytidine kinase	P27707	-0.5	0.0014
DCN	Decorin	P07585	-0.7	0.0496
DCTPP1	dCTP pyrophosphatase 1	Q9H773	-0.6	0.0072
DCXR	L-xylulose reductase	Q7Z4W1	0.8	<0.0001
DDAH2	N(G),N(G)-dimethylarginine dimethylaminohydrolase 2	O95865	0.6	0.0063
DDX18	ATP-dependent RNA helicase DDX18	Q9NVP1	-0.9	<0.0001
DDX23	Probable ATP-dependent RNA helicase DDX23	Q9BUQ8	-0.4	0.0005
DDX24	ATP-dependent RNA helicase DDX24	Q9GZR7	-0.5	0.0407
DDX27	Probable ATP-dependent RNA helicase DDX27	Q96GQ7	-0.5	0.0036
DDX42	ATP-dependent RNA helicase DDX42	Q86XP3-2;Q86XP3	-0.3	<0.0001
DDX47	Probable ATP-dependent RNA helicase DDX47	Q9H0S4-2;Q9H0S4	-0.6	0.0140
DDX6	Probable ATP-dependent RNA helicase DDX6	P26196	-0.3	0.0338
DENR	Density-regulated protein	O43583	-0.6	0.0388
DGKZ	Diacylglycerol kinase zeta	Q13574-6;Q13574-2;Q13574-4;Q13574-7;Q13574-5;Q13574-3;Q13574	0.4	0.0165
DHX30	Putative ATP-dependent RNA helicase DHX30	Q7L2E3-3;Q7L2E3;Q7L2E3-2	-0.3	0.0184
DHX9	ATP-dependent RNA helicase A	Q08211	-0.2	0.0001
DIAPH1	Protein diaphanous homolog 1	O60610-2;O60610-3;O60610	0.3	0.0018
DLAT	Dihydrolipoyllysine-residue acetyltransferase component of pyruvate dehydrogenase complex, mitochondrial	P10515	-0.2	0.0323
DNAJA2	DnaJ homolog subfamily A member 2	O60884	-0.6	0.0031
DNAJC13	DnaJ homolog subfamily C member 13	O75165	0.5	<0.0001

DNAJC7	DnaJ homolog subfamily C member 7	Q99615-2;Q99615	-0.3	0.0263
DNPEP	Aspartyl aminopeptidase	Q9ULA0	0.6	<0.0001
DOCK2	Dedicator of cytokinesis protein 2	Q92608	0.2	0.0019
DOCK8	Dedicator of cytokinesis protein 8	Q8NF50-4;Q8NF50-3;Q8NF50-2;Q8NF50	-0.3	<0.0001
DOK2	Docking protein 2	O60496	1.4	0.0131
DPY30	Protein dpy-30 homolog	Q9C005	-0.6	0.0025
DPYD	Dihydropyrimidine dehydrogenase [NADP(+)]	Q12882	0.6	0.0046
DPYSL2	Dihydropyrimidinase-related protein 2	Q16555;Q16555-2	0.3	0.0090
DSP	Desmoplakin	P15924	1.7	0.0098
DUSP3	Dual specificity protein phosphatase 3	P51452-2;P51452	0.6	0.0087
DYNC1H1	Cytoplasmic dynein 1 heavy chain 1	Q14204	0.2	<0.0001
DYSF	Dysferlin	O75923-15;O75923-3;O75923-9;O75923;O75923-14;O75923-6;O75923-12;O75923-5;O75923-11;O75923-4;O75923-10;O75923-2;O75923-8;O75923-7;O75923-13	0.9	0.0002
EBI3	Interleukin-27 subunit beta	Q14213	1.8	0.0059
EBNA1BP2	Probable rRNA-processing protein EBP2	Q99848	-0.4	0.0349
ECI1	Enoyl-CoA delta isomerase 1, mitochondrial	P42126	1.2	0.0209
ECM29	Proteasome-associated protein ECM29 homolog	Q5VYK3	0.3	0.0004
EEA1	Early endosome antigen 1	Q15075	-0.3	0.0312
EFHD2	EF-hand domain-containing protein D2	Q96C19	0.5	<0.0001
EHD1	EH domain-containing protein 1	Q9H4M9	0.3	0.0347
EIF2S2	Eukaryotic translation initiation factor 2 subunit 2	P20042	-0.2	0.0380
EIF3B	Eukaryotic translation initiation factor 3 subunit B	P55884;P55884-2	-0.2	0.0459
EIF3D	Eukaryotic translation initiation factor 3 subunit D	O15371-2;O15371-3;O15371	-0.2	0.0312
EIF3E	Eukaryotic translation initiation factor 3 subunit E	P60228	-0.3	0.0020
EIF3G	Eukaryotic translation initiation factor 3 subunit G	O75821	-0.3	0.0216

ELMO2	Engulfment and cell motility protein 2	Q96JJ3-3;Q96JJ3	0.5	0.0384
EMG1	Ribosomal RNA small subunit methyltransferase NEP1	Q92979	-0.7	0.0008
EMILIN1	EMILIN-1	Q9Y6C2	0.4	0.0150
EPRS	Bifunctional glutamate/proline--tRNA ligase;Glutamate--tRNA ligase;Proline--tRNA ligase	P07814	-0.2	0.0009
ERO1L	ERO1-like protein alpha	Q96HE7	0.4	0.0002
ERP29	Endoplasmic reticulum resident protein 29	P30040	0.4	0.0059
ETHE1	Persulfide dioxygenase ETHE1, mitochondrial	O95571	0.5	0.0085
EZR	Ezrin	P15311	-0.6	<0.0001
F2	Activation peptide fragment 1;Activation peptide fragment 2;Prothrombin;Thrombin heavy chain;Thrombin light chain	P00734	0.5	0.0379
FAM107B	Protein FAM107B	Q9H098;Q9H098-2	-0.6	0.0371
FAM129A	Protein Niban	Q9BZQ8	0.4	0.0452
FAM26F	Protein FAM26F	Q5R3K3	1.8	0.0108
FAM3C	Protein FAM3C	Q92520	-0.9	0.0018
FAM98A	Protein FAM98A	Q8NCA5-2;Q8NCA5	-1.1	0.0001
FASN	[Acyl-carrier-protein] S-acetyltransferase;[Acyl-carrier-protein] S-malonyltransferase;3-hydroxyacyl-[acyl-carrier-protein] dehydratase;3-oxoacyl-[acyl-carrier-protein] reductase;3-oxoacyl-[acyl-carrier-protein] synthase;Enoyl-[acyl-carrier-protein] reductase;Fatty acid synthase;Oleoyl-[acyl-carrier-protein] hydrolase	P49327	-0.2	0.0102
FBL	rRNA 2'-O-methyltransferase fibrillarin	P22087	-0.4	0.0057
FBLN1	Fibulin-1	P23142-4	-0.8	0.0216
FBLN5	Fibulin-5	Q9UBX5	-1.0	0.0396
FBN1	Fibrillin-1	P35555	1.0	<0.0001
FBP1	Fructose-1,6-bisphosphatase 1	P09467	0.6	0.0314

FCER1G	High affinity immunoglobulin epsilon receptor subunit gamma	P30273	1.8	0.0114
FCGR1B;FCGR1A;FCGR1C	High affinity immunoglobulin gamma Fc receptor I;High affinity immunoglobulin gamma Fc receptor IB;Putative high affinity immunoglobulin gamma Fc receptor IC	Q92637;P12314;A6NKC4;Q92637-3	1.5	0.0196
FGA	Fibrinogen alpha chain;Fibrinopeptide A	P02671-2;P02671	1.2	<0.0001
FGB	Fibrinogen beta chain;Fibrinopeptide B	P02675	1.6	<0.0001
FGG	Fibrinogen gamma chain	P02679-2;P02679	1.8	<0.0001
FKBP4	Peptidyl-prolyl cis-trans isomerase FKBP4;Peptidyl-prolyl cis-trans isomerase FKBP4, N-terminally processed	Q02790	-0.3	0.0320
FLNA	Filamin-A	P21333-2;P21333	-0.3	<0.0001
FMNL1	Formin-like protein 1	O95466;O95466-2;O95466-3	0.3	0.0338
FNBP1	Formin-binding protein 1	Q96RU3-4;Q96RU3-3;Q96RU3-5;Q96RU3-2;Q96RU3	-0.6	0.0040
FSCN1	Fascin	Q16658	-0.4	0.0109
FTH1	Ferritin heavy chain;Ferritin heavy chain, N-terminally processed	P02794	0.6	0.0062
FTL	Ferritin light chain	P02792	0.7	0.0022
FTSJ3	pre-rRNA processing protein FTSJ3	Q8IY81	-0.7	0.0004
FUBP1	Far upstream element-binding protein 1	Q96AE4;Q96AE4-2	-0.2	0.0418
FYB	FYN-binding protein	O15117;O15117-2;O15117-3	0.5	0.0015
GAA	70 kDa lysosomal alpha-glucosidase;76 kDa lysosomal alpha-glucosidase;Lysosomal alpha-glucosidase	P10253	0.4	0.0202
GALK1	Galactokinase	P51570;P51570-2	0.4	0.0071
GALM	Aldose 1-epimerase	Q96C23	0.9	0.0059
GBP1	Interferon-induced guanylate-binding protein 1	P32455	0.9	<0.0001
GBP2	Interferon-induced guanylate-binding protein 2	P32456	1.2	<0.0001

GCA	Grancalcin	P28676	0.6	0.0022
GEMIN4	Gem-associated protein 4	P57678	-0.5	0.0192
GGA2	ADP-ribosylation factor-binding protein GGA2	Q9UJY4	-0.6	0.0001
GIMAP1	GTPase IMAP family member 1	Q8WWP7	0.6	0.0041
GIMAP4	GTPase IMAP family member 4	Q9NUV9	0.9	<0.0001
GIMAP5	GTPase IMAP family member 5	Q96F15;Q96F15-2	0.8	0.0041
GIMAP7	GTPase IMAP family member 7	Q8NHV1	1.3	0.0375
GLIPR2	Golgi-associated plant pathogenesis-related protein 1	Q9H4G4	0.6	0.0107
GM2A	Ganglioside GM2 activator;Ganglioside GM2 activator isoform short	P17900	0.6	0.0064
GMPPA	Mannose-1-phosphate guanyltransferase alpha	Q96IJ6;Q96IJ6-2	0.5	<0.0001
GMPPB	Mannose-1-phosphate guanyltransferase beta	Q9Y5P6;Q9Y5P6-2	0.3	0.0352
GNAI2	Guanine nucleotide-binding protein G(i) subunit alpha-2	P04899;P04899-6;P04899-3;P04899-5;P04899-2;P04899-4	0.7	<0.0001
GNL3	Guanine nucleotide-binding protein-like 3	Q9BVP2-2;Q9BVP2	-0.7	0.0009
GP1BB	Platelet glycoprotein Ib beta chain	P13224;P13224-2	-3.2	0.0433
GPX1	Glutathione peroxidase 1	P07203	0.7	<0.0001
GRAP2	GRB2-related adapter protein 2	O75791	1.2	0.0169
GSS	Glutathione synthetase	P48637	0.5	0.0163
GTF2I	General transcription factor II-I	P78347-2;P78347-4;P78347-3;P78347	-0.4	<0.0001
GTPBP10	GTP-binding protein 10	A4D1E9-2;A4D1E9	-0.8	0.0381
GTPBP4	Nucleolar GTP-binding protein 1	Q9BZE4	-0.7	0.0077
GZMB	Granzyme B	P10144	1.3	0.0373
GZMH	Granzyme H	P20718	2.7	0.0282
GZMK	Granzyme K	P49863	0.6	0.0172
H2AFY	Core histone macro-H2A.1	O75367-2;O75367-3;O75367	-0.2	0.0366
H6PD	6-phosphogluconolactonase;GDH/6PGL endoplasmic bifunctional protein;Glucose 1-dehydrogenase	O95479	0.7	0.0077

HACD4	Very-long-chain (3R)-3-hydroxyacyl-CoA dehydratase 4	Q5VWC8	-1.1	0.0201
HADHA	Long chain 3-hydroxyacyl-CoA dehydrogenase;Long-chain enoyl-CoA hydratase;Trifunctional enzyme subunit alpha, mitochondrial	P40939	0.3	0.0001
HCK	Tyrosine-protein kinase HCK	P08631-3;P08631-2;P08631-4;P08631	0.6	0.0177
HEATR1	HEAT repeat-containing protein 1;HEAT repeat-containing protein 1, N-terminally processed	Q9H583	-0.4	<0.0001
HK3	Hexokinase-3	P52790	1.7	<0.0001
HLA-DOA	HLA class II histocompatibility antigen, DO alpha chain	P06340	-1.1	0.0286
HLA-DPB1	HLA class II histocompatibility antigen, DP beta 1 chain	P04440	-1.0	0.0002
HLA-DQB1	HLA class II histocompatibility antigen, DQ beta 1 chain	P01920	-0.8	0.0190
HSD11B1	Corticosteroid 11-beta-dehydrogenase isozyme 1	P28845	1.5	0.0319
HSDL1	Inactive hydroxysteroid dehydrogenase-like protein 1	Q3SXM5-2;Q3SXM5	-0.6	0.0302
HSP90B1	Endoplasmin	P14625	0.2	0.0120
HSPA14	Heat shock 70 kDa protein 14	Q0VDF9	-0.3	0.0497
HSPD1	60 kDa heat shock protein, mitochondrial	P10809	-0.3	0.0001
HSPE1	10 kDa heat shock protein, mitochondrial	P61604	-0.5	0.0004
HSPG2	Basement membrane-specific heparan sulfate proteoglycan core protein;Endorepellin;LG3 peptide	P98160	-0.4	<0.0001
HTATSF1	HIV Tat-specific factor 1	O43719	-0.6	0.0274
HUWE1	E3 ubiquitin-protein ligase HUWE1	Q7Z6Z7-2;Q7Z6Z7-3;Q7Z6Z7	-0.1	0.0214
IARS	Isoleucine--tRNA ligase, cytoplasmic	P41252	-0.3	<0.0001
IDH1	Isocitrate dehydrogenase [NADP] cytoplasmic	O75874	0.5	<0.0001
IDO1	Indoleamine 2,3-dioxygenase 1	P14902	2.9	<0.0001
IFI35	Interferon-induced 35 kDa protein	P80217;P80217-2	1.5	<0.0001

IGF2R	Cation-independent mannose-6-phosphate receptor	P11717	0.6	<0.0001
IMPDH2	Inosine-5'-monophosphate dehydrogenase 2	P12268	-0.4	0.0011
INPP5D	Phosphatidylinositol 3,4,5-trisphosphate 5-phosphatase 1	Q92835-2;Q92835	-0.4	0.0045
INTS1	Integrator complex subunit 1	Q8N201	-0.4	0.0356
IQGAP1	Ras GTPase-activating-like protein IQGAP1	P46940	0.4	<0.0001
IQGAP2	Ras GTPase-activating-like protein IQGAP2	Q13576	0.4	0.0016
IQSEC1	IQ motif and SEC7 domain-containing protein 1	Q6DN90-2;Q6DN90	-0.6	0.0322
ISG15	Ubiquitin-like protein ISG15	P05161	-0.8	0.0416
ISLR	Immunoglobulin superfamily containing leucine-rich repeat protein	O14498	-2.2	0.0041
ITGAX	Integrin alpha-X	P20702	-0.6	<0.0001
IVL	Involucrin	P07476	2.5	0.0062
KCTD12	BTB/POZ domain-containing protein KCTD12	Q96CX2	0.5	0.0028
KDM1A	Lysine-specific histone demethylase 1A	O60341;O60341-2	-0.4	0.0170
KHSRP	Far upstream element-binding protein 2	Q92945	-0.2	0.0406
KIAA0020	Pumilio domain-containing protein KIAA0020	Q15397	-0.5	0.0095
KIF13B	Kinesin-like protein KIF13B	Q9NQT8	0.8	0.0412
KLHL14	Kelch-like protein 14	Q9P2G3-2;Q9P2G3	2.1	0.0237
KPNA2	Importin subunit alpha-1	P52292	-0.3	0.0380
KPNB1	Importin subunit beta-1	Q14974	-0.2	0.0298
LACTB	Serine beta-lactamase-like protein LACTB, mitochondrial	P83111	0.8	0.0039
LACTB2	Beta-lactamase-like protein 2	Q53H82	-0.9	0.0323
LAP3	Cytosol aminopeptidase	P28838-2;P28838	0.4	<0.0001
LASP1	LIM and SH3 domain protein 1	Q14847;Q14847-2	-0.6	0.0110
LBP	Lipopolysaccharide-binding protein	P18428	1.1	0.0059
LBR	Lamin-B receptor	Q14739	-0.4	0.0031
LCP2	Lymphocyte cytosolic protein 2	Q13094	0.9	<0.0001

LMAN1	Protein ERGIC-53	P49257	-0.3	0.0421
LMAN2	Vesicular integral-membrane protein VIP36	Q12907	0.3	0.0018
LPCAT1	Lysophosphatidylcholine acyltransferase 1	Q8NF37	-0.4	0.0037
LRG1	Leucine-rich alpha-2-glycoprotein	P02750	0.9	0.0017
LRMP	Lymphoid-restricted membrane protein;Processed lymphoid-restricted membrane protein	Q12912-2;Q12912	-0.8	<0.0001
LRP1	Low-density lipoprotein receptor-related protein 1 515 kDa subunit;Low-density lipoprotein receptor-related protein 1 85 kDa subunit;Low-density lipoprotein receptor-related protein 1 intracellular domain;Prolow-density lipoprotein receptor-related protein 1	Q07954	0.6	<0.0001
LRPPRC	Leucine-rich PPR motif-containing protein, mitochondrial	P42704	-0.4	<0.0001
LRRC25	Leucine-rich repeat-containing protein 25	Q8N386	1.2	0.0407
LRRC40	Leucine-rich repeat-containing protein 40	Q9H9A6	-0.6	<0.0001
LRRK2	Leucine-rich repeat serine/threonine-protein kinase 2	Q5S007	1.2	0.0027
LSM1	U6 snRNA-associated Sm-like protein LSm1	O15116	0.5	0.0444
LTA4H	Leukotriene A-4 hydrolase	P09960;P09960-2	0.7	0.0022
LTBP2	Latent-transforming growth factor beta-binding protein 2	Q14767	-1.0	<0.0001
LUM	Lumican	P51884	-0.9	0.0011
LXN	Latexin	Q9BS40	-1.4	0.0151
LYZ	Lysozyme C	P61626	0.6	0.0083
MALT1	Mucosa-associated lymphoid tissue lymphoma translocation protein 1	Q9UDY8-2;Q9UDY8	-0.8	0.0278
MAP4K1	Mitogen-activated protein kinase kinase kinase kinase 1	Q92918-2;Q92918	-0.3	0.0411
MBD2	Methyl-CpG-binding domain protein 2	Q9UBB5	-0.7	0.0053

MCCC1	Methylcrotonoyl-CoA carboxylase subunit alpha, mitochondrial	Q96RQ3	-0.7	0.0044
MCM2	DNA replication licensing factor MCM2	P49736	-0.3	0.0068
MCM4	DNA replication licensing factor MCM4	P33991	-0.3	0.0079
MCM5	DNA replication licensing factor MCM5	P33992	-0.3	0.0017
MCM6	DNA replication licensing factor MCM6	Q14566	-0.4	0.0004
MCM7	DNA replication licensing factor MCM7	P33993;P33993-3	-0.4	0.0014
ME1	NADP-dependent malic enzyme	P48163-2;P48163	1.1	0.0020
ME2	NAD-dependent malic enzyme, mitochondrial	P23368	-0.4	0.0375
METTL7A	Methyltransferase-like protein 7A	Q9H8H3	0.4	0.0151
MGST3	Microsomal glutathione S-transferase 3	O14880	0.6	0.0239
MNDA	Myeloid cell nuclear differentiation antigen	P41218	0.7	0.0028
MPST	3-mercaptopyruvate sulfurtransferase	P25325;P25325-2	0.5	0.0111
MRC2	C-type mannose receptor 2	Q9UBG0	-0.7	0.0352
MRPL1	39S ribosomal protein L1, mitochondrial	Q9BYD6	-0.4	0.0175
MRPL12	39S ribosomal protein L12, mitochondrial	P52815	-0.6	0.0364
MRPL15	39S ribosomal protein L15, mitochondrial	Q9P015	-0.6	0.0216
MRPL19	39S ribosomal protein L19, mitochondrial	P49406	-0.4	0.0418
MRPL22	39S ribosomal protein L22, mitochondrial	Q9NWU5-2;Q9NWU5-3;Q9NWU5	-0.4	0.0243
MRPL43	39S ribosomal protein L43, mitochondrial	Q8N983-4;Q8N983-3;Q8N983-2;Q8N983;Q8N983-6;Q8N983-7	-0.5	0.0184
MRPL49	39S ribosomal protein L49, mitochondrial	Q13405	-0.6	0.0271
MRPS6	28S ribosomal protein S6, mitochondrial	P82932	-1.0	0.0148
MT-CO2	Cytochrome c oxidase subunit 2	P00403	0.4	0.0168
MTHFD1	C-1-tetrahydrofolate synthase, cytoplasmic;C-1-tetrahydrofolate synthase, cytoplasmic, N-terminally processed;Formyltetrahydrofolate synthetase;Methenyltetrahydrofolate cyclohydrolase;Methylenetetrahydrofolate dehydrogenase	P11586	-0.2	0.0021

MVP	Major vault protein	Q14764	0.4	<0.0001
MX2	Interferon-induced GTP-binding protein Mx2	P20592	-1.2	0.0041
MXRA5	Matrix-remodeling-associated protein 5	Q9NR99	0.7	0.0071
MYBBP1A	Myb-binding protein 1A	Q9BQG0;Q9BQG0-2	-0.4	<0.0001
MYCBP2	E3 ubiquitin-protein ligase MYCBP2	O75592-2;O75592	-0.6	0.0022
MYH11	Myosin-11	P35749-4;P35749-3;P35749;P35749-2	-1.0	0.0021
MYO1D	Unconventional myosin-Id	O94832	-0.7	0.0151
MYO1F	Unconventional myosin-If	O00160	0.8	<0.0001
MYO1G	Minor histocompatibility antigen HA-2;Unconventional myosin-Ig	B0I1T2	0.4	0.0012
MYOF	Myoferlin	Q9NZM1-6;Q9NZM1;Q9NZM1-2;Q9NZM1-5	1.7	0.0001
MYOF	Myoferlin	Q9NZM1-6;Q9NZM1;Q9NZM1-3;Q9NZM1-2	1.6	<0.0001
NAGK	N-acetyl-D-glucosamine kinase	Q9UJ70;Q9UJ70-2	0.7	<0.0001
NAMPT	Nicotinamide phosphoribosyltransferase	P43490	0.3	0.0356
NCAPD2	Condensin complex subunit 1	Q15021	-0.4	0.0002
NCAPG	Condensin complex subunit 3	Q9BPX3	-0.5	0.0169
NCL	Nucleolin	P19338	-0.4	<0.0001
NFKB2	Nuclear factor NF-kappa-B p100 subunit;Nuclear factor NF-kappa-B p52 subunit	Q00653-4;Q00653	0.4	0.0042
NOB1	RNA-binding protein NOB1	Q9ULX3	-0.8	0.0017
NOLC1	Nucleolar and coiled-body phosphoprotein 1	Q14978-3	-0.7	<0.0001
NOP56	Nucleolar protein 56	O00567	-0.2	0.0102
NPL	N-acetylneuraminate lyase	Q9BXD5;Q9BXD5-2	0.7	0.0340
NT5C3A	Cytosolic 5'-nucleotidase 3A	Q9H0P0-3;Q9H0P0-2;Q9H0P0-1;Q9H0P0	-0.6	0.0313
NTPCR	Cancer-related nucleoside-triphosphatase	Q9BSD7	-0.5	0.0061
NUCB1	Nucleobindin-1	Q02818	0.4	0.0088

NUDT21	Cleavage and polyadenylation specificity factor subunit 5	O43809	-0.2	0.0041
NUP133	Nuclear pore complex protein Nup133	Q8WUM0	-0.2	0.0412
NUP155	Nuclear pore complex protein Nup155	O75694-2;O75694	-0.2	0.0068
NUP205	Nuclear pore complex protein Nup205	Q92621	-0.3	<0.0001
NUP37	Nucleoporin Nup37	Q8NFH4	-0.5	0.0173
OGN	Mimecan	P20774	-1.2	<0.0001
OLFM4	Olfactomedin-4	Q6UX06	-3.7	0.0035
OLFML1	Olfactomedin-like protein 1	Q6UWY5	-1.2	0.0047
OPA1	Dynamin-like 120 kDa protein, form S1;Dynamin-like 120 kDa protein, mitochondrial	O60313;O60313-2	-0.3	0.0032
ORM1	Alpha-1-acid glycoprotein 1	P02763	1.3	0.0002
OSBP	Oxysterol-binding protein 1	P22059	-0.3	0.0125
OSTF1	Osteoclast-stimulating factor 1	Q92882	0.4	0.0023
PACSIN2	Protein kinase C and casein kinase substrate in neurons protein 2	Q9UNF0-2;Q9UNF0	0.4	0.0003
PARK7	Protein deglycase DJ-1	Q99497	0.3	0.0375
PARN	Poly(A)-specific ribonuclease PARN	O95453-4;O95453-2;O95453-3;O95453	-0.7	0.0286
PARP1	Poly [ADP-ribose] polymerase 1	P09874	-0.5	<0.0001
PARP14	Poly [ADP-ribose] polymerase 14	Q460N5-4;Q460N5-1;Q460N5	0.6	0.0028
PARP4	Poly [ADP-ribose] polymerase 4	Q9UKK3	0.5	0.0036
PARP9	Poly [ADP-ribose] polymerase 9	Q8IXQ6-3;Q8IXQ6-2;Q8IXQ6	0.5	0.0020
PBDC1	Protein PBDC1	Q9BVG4	-0.5	0.0190
PCNP	PEST proteolytic signal-containing nuclear protein	Q8WW12;Q8WW12-2	-0.8	0.0407
PDAP1	28 kDa heat- and acid-stable phosphoprotein	Q13442	-0.5	0.0162
PDCD10	Programmed cell death protein 10	Q9BUL8	-0.4	0.0314
PDCD11	Protein RRP5 homolog	Q14690	-0.5	<0.0001
PDS5B	Sister chromatid cohesion protein PDS5 homolog B	Q9NTI5-2;Q9NTI5	-0.3	0.0111

PELP1	Proline-, glutamic acid- and leucine-rich protein 1	Q8IZL8	-0.4	0.0002
PES1	Pescadillo homolog	O00541-2;O00541	-0.6	0.0001
PFAS	Phosphoribosylformylglycinamidine synthase	O15067	-0.5	<0.0001
PFDN1	Prefoldin subunit 1	O60925	-0.7	0.0003
PFDN2	Prefoldin subunit 2	Q9UHV9	-0.8	0.0103
PFDN5	Prefoldin subunit 5	Q99471;Q99471-3	-0.5	0.0062
PFDN6	Prefoldin subunit 6	O15212	-0.6	0.0103
PFKM	ATP-dependent 6-phosphofructokinase, muscle type	P08237;P08237-3;P08237-2	-0.6	0.0274
PGLS	6-phosphogluconolactonase	O95336	0.4	0.0001
PGM2	Phosphoglucomutase-2	Q96G03	0.4	0.0140
PHB2	Prohibitin-2	Q99623	-0.7	0.0014
PHF5A	PHD finger-like domain-containing protein 5A	Q7RTV0	-0.7	0.0014
PHGDH	D-3-phosphoglycerate dehydrogenase	O43175	-0.5	0.0074
PLA2G4A	Cytosolic phospholipase A2;Lysophospholipase;Phospholipase A2	P47712	1.8	0.0114
PLCG2	1-phosphatidylinositol 4,5-bisphosphate phosphodiesterase gamma-2	P16885	-0.3	<0.0001
PLEK	Pleckstrin	P08567	0.5	0.0024
PLG	Activation peptide;Angiostatin;Plasmin heavy chain A;Plasmin heavy chain A, short form;Plasmin light chain B;Plasminogen	P00747	0.6	<0.0001
PLOD3	Procollagen-lysine,2-oxoglutarate 5-dioxygenase 3	O60568	0.6	0.0019
PLSCR1	Phospholipid scramblase 1	O15162-2;O15162	0.6	0.0322
PML	Protein PML	P29590;P29590-11	0.9	0.0310
PMPCB	Mitochondrial-processing peptidase subunit beta	O75439	-0.3	0.0332
PNPT1	Polyribonucleotide nucleotidyltransferase 1, mitochondrial	Q8TCS8	-0.5	0.0001
POGZ	Pogo transposable element with ZNF domain	Q7Z3K3-5;Q7Z3K3-7;Q7Z3K3-2;Q7Z3K3-3;Q7Z3K3-6;Q7Z3K3	-0.3	0.0380

POLR1C	DNA-directed RNA polymerases I and III subunit RPAC1	O15160-2;O15160	-0.4	0.0312
POR	NADPH--cytochrome P450 reductase	P16435	-0.4	0.0097
PPA1	Inorganic pyrophosphatase	Q15181	0.8	<0.0001
PPAN	Suppressor of SWI4 1 homolog	Q9NQ55-2;Q9NQ55;Q9NQ55-3	-0.7	0.0429
PPAT	Amidophosphoribosyltransferase	Q06203	-0.5	0.0002
PPP1R9B	Neurabin-2	Q96SB3	-0.4	0.0018
PPP2R4	Serine/threonine-protein phosphatase 2A activator	Q15257-3;Q15257-2;Q15257	0.5	0.0019
PREX1	Phosphatidylinositol 3,4,5-trisphosphate-dependent Rac exchanger 1 protein	Q8TCU6;Q8TCU6-2	0.8	0.0062
PRKAR1A	cAMP-dependent protein kinase type I-alpha regulatory subunit;cAMP-dependent protein kinase type I-alpha regulatory subunit, N-terminally processed	P10644	0.8	0.0211
PRKCB	Protein kinase C beta type	P05771-2	0.9	0.0323
PRMT1	Protein arginine N-methyltransferase 1	Q99873-3;Q99873-2;Q99873-4;Q99873	-0.6	<0.0001
PRPF19	Pre-mRNA-processing factor 19	Q9UMS4	-0.3	0.0155
PRPF3	U4/U6 small nuclear ribonucleoprotein Prp3	O43395	-0.5	0.0338
PRPF8	Pre-mRNA-processing-splicing factor 8	Q6P2Q9	-0.2	0.0018
PRPSAP1	Phosphoribosyl pyrophosphate synthase-associated protein 1	Q14558;Q14558-2	0.4	0.0022
PSMA4	Proteasome subunit alpha type-4	P25789	0.5	0.0394
PSMB1	Proteasome subunit beta type-1	P20618	0.3	0.0493
PSMB10	Proteasome subunit beta type-10	P40306	0.7	0.0060
PSMB3	Proteasome subunit beta type-3	P49720	0.5	0.0274
PSMB6	Proteasome subunit beta type-6	P28072	-1.3	<0.0001
PSMB9	Proteasome subunit beta type-9	P28065-2;P28065	0.7	0.0021
PSMD10	26S proteasome non-ATPase regulatory subunit 10	O75832	-0.6	0.0216
PSMD3	26S proteasome non-ATPase regulatory subunit 3	O43242	-0.3	0.0270

PSME2	Proteasome activator complex subunit 2	Q9UL46	0.6	<0.0001
PSME3	Proteasome activator complex subunit 3	P61289;P61289-3;P61289-2	-0.5	0.0069
PSPC1	Paraspeckle component 1	Q8WXF1-2;Q8WXF1	-0.4	0.0002
PTCD3	Pentatricopeptide repeat domain-containing protein 3, mitochondrial	Q96EY7	-0.5	0.0282
PTER	Phosphotriesterase-related protein	Q96BW5-2;Q96BW5	1.1	0.0363
PTPN1	Tyrosine-protein phosphatase non-receptor type 1	P18031	0.5	0.0004
PTPRE	Receptor-type tyrosine-protein phosphatase epsilon	P23469-3;P23469-2;P23469	0.7	0.0078
PURA	Transcriptional activator protein Pur-alpha	Q00577	0.3	0.0457
PWP2	Periodic tryptophan protein 2 homolog	Q15269	-0.6	0.0444
PXDN	Peroxidasin homolog	Q92626	-0.7	0.0171
PYCARD	Apoptosis-associated speck-like protein containing a CARD	Q9ULZ3;Q9ULZ3-2	0.7	0.0352
PYGB	Glycogen phosphorylase, brain form	P11216	0.6	<0.0001
PYGL	Glycogen phosphorylase, liver form	P06737-2;P06737	1.1	0.0014
RAB21	Ras-related protein Rab-21	Q9UL25	-0.4	0.0302
RAB3GAP2	Rab3 GTPase-activating protein non-catalytic subunit	Q9H2M9	-0.4	<0.0001
RAD23B	UV excision repair protein RAD23 homolog B	P54727	-0.9	0.0061
RAE1	mRNA export factor	P78406	-0.4	<0.0001
RAN	GTP-binding nuclear protein Ran	P62826	-0.3	0.0235
RANBP1	Ran-specific GTPase-activating protein	P43487-2;P43487	-0.4	0.0286
RANBP2	E3 SUMO-protein ligase RanBP2	P49792	-0.3	0.0041
RANGAP1	Ran GTPase-activating protein 1	P46060	-0.2	0.0421
RBM10	RNA-binding protein 10	P98175-4;P98175-3;P98175-2;P98175	-0.3	0.0356
RBM14	RNA-binding protein 14	Q96PK6	-0.2	0.0134
RBM28	RNA-binding protein 28	Q9NW13-2;Q9NW13	-0.5	0.0320
RBP4	Plasma retinol-binding protein(1-176);Plasma retinol-binding protein(1-179);Plasma retinol-binding	P02753	-0.6	0.0429

	protein(1-181);Plasma retinol-binding protein(1-182);Retinol-binding protein 4			
RCC1	Regulator of chromosome condensation	P18754;P18754-2	-0.6	<0.0001
REL	Proto-oncogene c-Rel	Q04864-2;Q04864	-0.7	<0.0001
RENBP	N-acylglucosamine 2-epimerase	P51606	0.6	0.0277
RFTN1	Raftlin	Q14699	-0.8	<0.0001
RMDN1	Regulator of microtubule dynamics protein 1	Q96DB5-2;Q96DB5	-0.5	0.0319
RNASE3	Eosinophil cationic protein	P12724	-1.3	0.0072
RNF213	E3 ubiquitin-protein ligase RNF213	Q63HN8;Q63HN8-4	0.8	<0.0001
RNH1	Ribonuclease inhibitor	P13489	0.6	<0.0001
RNMT	mRNA cap guanine-N7 methyltransferase	O43148;O43148-2	-0.6	0.0184
RNPEP	Aminopeptidase B	Q9H4A4	0.5	<0.0001
RPL23A	60S ribosomal protein L23a	P62750	-0.4	0.0352
RPL27	60S ribosomal protein L27	P61353	-0.3	0.0175
RPL38	60S ribosomal protein L38	P63173	-0.5	0.0320
RPL4	60S ribosomal protein L4	P36578	-0.4	<0.0001
RPL6	60S ribosomal protein L6	Q02878	-0.3	0.0036
RPL7	60S ribosomal protein L7	P18124	-0.2	0.0312
RPL7A	60S ribosomal protein L7a	P62424	-0.3	<0.0001
RPRD1A	Regulation of nuclear pre-mRNA domain-containing protein 1A	Q96P16;Q96P16-3	-1.4	0.0312
RPS12	40S ribosomal protein S12	P25398	-0.4	0.0088
RPS16	40S ribosomal protein S16	P62249	-0.3	0.0071
RPS18	40S ribosomal protein S18	P62269	-0.3	0.0111
RPS19	40S ribosomal protein S19	P39019	-0.3	0.0154
RPS2	40S ribosomal protein S2	P15880	-0.2	0.0343
RPS20	40S ribosomal protein S20	P60866;P60866-2	-0.3	0.0002
RPS25	40S ribosomal protein S25	P62851	-0.4	0.0102

RPS3A	40S ribosomal protein S3a	P61247	-0.3	0.0036
RPS6	40S ribosomal protein S6	P62753	-0.3	0.0433
RPS9	40S ribosomal protein S9	P46781	-0.3	0.0068
RRAS	Ras-related protein R-Ras	P10301	0.6	0.0179
RRM1	Ribonucleoside-diphosphate reductase large subunit	P23921	-0.7	0.0144
RRP1	Ribosomal RNA processing protein 1 homolog A	P56182	-0.6	<0.0001
RRP15	RRP15-like protein	Q9Y3B9	-0.5	0.0177
RRP9	U3 small nucleolar RNA-interacting protein 2	O43818	-0.5	0.0018
RSL1D1	Ribosomal L1 domain-containing protein 1	O76021	-0.5	<0.0001
S100A11	Protein S100-A11;Protein S100-A11, N-terminally processed	P31949	0.5	0.0073
S100A4	Protein S100-A4	P26447	0.9	0.0028
S100A8	Protein S100-A8;Protein S100-A8, N-terminally processed	P05109	1.7	<0.0001
S100A9	Protein S100-A9	P06702	1.9	<0.0001
SAMSN1	SAM domain-containing protein SAMSN-1	Q9NSI8;Q9NSI8-3	-2.7	0.0154
SAP30BP	SAP30-binding protein	Q9UHR5-2;Q9UHR5	-0.7	0.0223
SART1	U4/U6.U5 tri-snRNP-associated protein 1	O43290	-0.2	0.0357
SCP2	Non-specific lipid-transfer protein	P22307-6;P22307-2;P22307-4;P22307-7;P22307-8;P22307	-0.3	0.0301
SDPR	Serum deprivation-response protein	O95810	-1.3	0.0051
SERPINA1	Alpha-1-antitrypsin;Short peptide from AAT	P01009	1.1	0.0069
SERPINA3	Alpha-1-antichymotrypsin;Alpha-1-antichymotrypsin His-Pro-less	P01011	0.7	0.0001
SERPINB1	Leukocyte elastase inhibitor	P30740	0.9	<0.0001
SERPINB6	Serpin B6	P35237	1.2	<0.0001
SERPINB9	Serpin B9	P50453	1.0	<0.0001
SERPIND1	Heparin cofactor 2	P05546	0.5	0.0209
SERPINF1	Pigment epithelium-derived factor	P36955	-0.6	0.0036

SERPING1	Plasma protease C1 inhibitor	P05155-2;P05155;P05155-3	1.0	<0.0001
SET	Protein SET	Q01105-2	-0.3	0.0472
SF3A1	Splicing factor 3A subunit 1	Q15459	-0.4	0.0147
SF3B1	Splicing factor 3B subunit 1	O75533	-0.3	<0.0001
SF3B2	Splicing factor 3B subunit 2	Q13435	-0.3	0.0094
SGPP1	Sphingosine-1-phosphate phosphatase 1	Q9BX95	-0.5	0.0361
SIGLEC1	Sialoadhesin	Q9BZZ2-2;Q9BZZ2-3;Q9BZZ2	0.9	0.0050
SLAMF1	Signaling lymphocytic activation molecule	Q13291-2;Q13291-3;Q13291;Q13291-4	-2.4	<0.0001
SLC25A12	Calcium-binding mitochondrial carrier protein Aralar1	O75746	-0.5	0.0339
SLC25A13	Calcium-binding mitochondrial carrier protein Aralar2	Q9UJS0;Q9UJS0-2	-0.4	0.0070
SLC29A1	Equilibrative nucleoside transporter 1	Q99808;Q99808-2	-0.6	0.0223
SLC2A6	Solute carrier family 2, facilitated glucose transporter member 6	Q9UGQ3-2;Q9UGQ3	1.2	0.0192
SLIRP	SRA stem-loop-interacting RNA-binding protein, mitochondrial	Q9GZT3-2;Q9GZT3	-0.5	0.0022
SMARCA5	SWI/SNF-related matrix-associated actin-dependent regulator of chromatin subfamily A member 5	O60264	-0.4	0.0001
SMC1A	Structural maintenance of chromosomes protein 1A	Q14683	-0.4	<0.0001
SMC3	Structural maintenance of chromosomes protein 3	Q9UQE7	-0.3	<0.0001
SMCHD1	Structural maintenance of chromosomes flexible hinge domain-containing protein 1	A6NHR9-2;A6NHR9	-0.4	0.0045
SND1	Staphylococcal nuclease domain-containing protein 1	Q7KZF4	-0.3	<0.0001
SNRNP200	U5 small nuclear ribonucleoprotein 200 kDa helicase	O75643	-0.2	0.0131
SNRPA1	U2 small nuclear ribonucleoprotein A'	P09661	-0.3	0.0018
SNX17	Sorting nexin-17	Q15036-2;Q15036	0.4	0.0123
SNX2	Sorting nexin-2	O60749-2;O60749	-0.2	0.0014

SOAT1	Sterol O-acyltransferase 1	P35610-3;P35610-2;P35610	0.9	0.0019
SOD1	Superoxide dismutase [Cu-Zn]	P00441	-0.4	0.0384
SOD2	Superoxide dismutase [Mn], mitochondrial	P04179;P04179-4;P04179-2	1.2	0.0005
SPN	Leukosialin	P16150	0.7	0.0306
SPTA1	Spectrin alpha chain, erythrocytic 1	P02549-2;P02549	-1.2	0.0008
SPTBN1	Spectrin beta chain, non-erythrocytic 1	Q01082	-0.6	0.0069
SQRDL	Sulfide:quinone oxidoreductase, mitochondrial	Q9Y6N5	0.7	<0.0001
SRPRB	Signal recognition particle receptor subunit beta	Q9Y5M8	-0.3	<0.0001
SRSF5	Serine/arginine-rich splicing factor 5	Q13243;Q13243-3	-0.4	0.0361
SRSF9	Serine/arginine-rich splicing factor 9	Q13242	-0.4	0.0155
SSRP1	FACT complex subunit SSRP1	Q08945	-0.2	0.0168
STAT5B	Signal transducer and activator of transcription 5B	P51692	0.6	0.0409
STAU1	Double-stranded RNA-binding protein Staufen homolog 1	O95793-2;O95793-3;O95793	-0.4	0.0237
STX11	Syntaxin-11	O75558	1.4	<0.0001
STX7	Syntaxin-7	O15400-2;O15400	-0.3	0.0470
STXBP2	Syntaxin-binding protein 2	Q15833-2;Q15833;Q15833-3	0.4	0.0003
SUCLG1	Succinyl-CoA ligase [ADP/GDP-forming] subunit alpha, mitochondrial	P53597	-0.5	0.0112
SUZ12	Polycomb protein SUZ12	Q15022	-0.5	0.0314
SWAP70	Switch-associated protein 70	Q9UH65	-0.6	<0.0001
SYNGR2	Synaptogyrin-2	O43760;O43760-2	-0.8	0.0018
TAGLN	Transgelin	Q01995	-0.8	<0.0001
TAP1	Antigen peptide transporter 1	Q03518	0.9	<0.0001
TAP2	Antigen peptide transporter 2	Q03519	0.9	0.0015
TAPBP	Tapasin	O15533-2;O15533;O15533-3	0.9	0.0212
TARS2	Threonine--tRNA ligase, mitochondrial	Q9BW92	-0.6	0.0120
TBC1D10C	Carabin	Q8IV04	0.3	0.0194
TBC1D9B	TBC1 domain family member 9B	Q66K14-2;Q66K14	0.4	0.0155

TCEB1	Transcription elongation factor B polypeptide 1	Q15369-2;Q15369	-0.4	0.0094
TCIRG1	V-type proton ATPase 116 kDa subunit a isoform 3	Q13488	0.6	0.0068
TCP1	T-complex protein 1 subunit alpha	P17987	-0.2	0.0116
TERF2	Telomeric repeat-binding factor 2	Q15554	-0.7	0.0038
TFRC	Transferrin receptor protein 1;Transferrin receptor protein 1, serum form	P02786	-0.4	0.0039
THEMIS2	Protein THEMIS2	Q5TEJ8	1.2	0.0150
THRAP3	Thyroid hormone receptor-associated protein 3	Q9Y2W1	-0.4	0.0002
TIMM50	Mitochondrial import inner membrane translocase subunit TIM50	Q3ZCQ8;Q3ZCQ8-2	-0.7	0.0085
TLN1	Talin-1	Q9Y490	0.1	0.0163
TMPO	Lamina-associated polypeptide 2, isoform alpha;Thymopentin;Thymopoietin	P42166	-0.5	0.0001
TMX1	Thioredoxin-related transmembrane protein 1	Q9H3N1	-0.3	0.0055
TNC	Tenascin	P24821;P24821-4	-0.7	0.0242
TNFAIP2	Tumor necrosis factor alpha-induced protein 2	Q03169	1.1	<0.0001
TNS3	Tensin-3	Q68CZ2	1.0	0.0338
TOMM22	Mitochondrial import receptor subunit TOM22 homolog	Q9NS69	-1.0	0.0030
TOMM70A	Mitochondrial import receptor subunit TOM70	O94826	-0.4	0.0411
TOP1	DNA topoisomerase 1	P11387	-0.6	<0.0001
TPM4	Tropomyosin alpha-4 chain	P67936	-0.4	0.0286
TPMT	Thiopurine S-methyltransferase	P51580	0.5	0.0185
TPP1	Tripeptidyl-peptidase 1	O14773-2;O14773	0.4	0.0423
TPP2	Tripeptidyl-peptidase 2	P29144	-0.2	0.0277
TPR	Nucleoprotein TPR	P12270	-0.3	<0.0001
TRAF1	TNF receptor-associated factor 1	Q13077-2;Q13077	-0.8	0.0394
TSR1	Pre-rRNA-processing protein TSR1 homolog	Q2NL82	-0.6	0.0018
TTR	Transthyretin	P02766	-0.8	<0.0001
TUFM	Elongation factor Tu, mitochondrial	P49411	-0.4	0.0004

TXNL1	Thioredoxin-like protein 1	O43396	-0.4	0.0037
U2SURP	U2 snRNP-associated SURP motif-containing protein	O15042-2;O15042	-0.5	0.0042
UBA7	Ubiquitin-like modifier-activating enzyme 7	P41226	0.8	<0.0001
UBE2J1	Ubiquitin-conjugating enzyme E2 J1	Q9Y385	-0.7	0.0271
UBTF	Nucleolar transcription factor 1	P17480-2;P17480	-0.5	0.0010
UCHL1	Ubiquitin carboxyl-terminal hydrolase isozyme L1	P09936	-1.4	0.0015
UPF1	Regulator of nonsense transcripts 1	Q92900-2;Q92900	-0.3	<0.0001
UQCRC2	Cytochrome b-c1 complex subunit 2, mitochondrial	P22695	-0.2	0.0275
URB1	Nucleolar pre-ribosomal-associated protein 1	O60287	-0.4	0.0233
USP15	Ubiquitin carboxyl-terminal hydrolase 15	Q9Y4E8-2;Q9Y4E8-3;Q9Y4E8	0.3	0.0416
UTP18	U3 small nucleolar RNA-associated protein 18 homolog	Q9Y5J1	-0.5	0.0281
UTP20	Small subunit processome component 20 homolog	O75691	-1.0	0.0169
UTP6	U3 small nucleolar RNA-associated protein 6 homolog	Q9NYH9	-0.4	0.0394
VAV1	Proto-oncogene vav	P15498-2;P15498	0.3	0.0441
VCAM1	Vascular cell adhesion protein 1	P19320-2;P19320-3;P19320	-0.5	0.0073
VCAN	Versican core protein	P13611-4;P13611-3;P13611-2;P13611-5;P13611	-1.0	0.0039
VCL	Vinculin	P18206-2;P18206	-0.6	<0.0001
VPS18	Vacuolar protein sorting-associated protein 18 homolog	Q9P253	0.4	0.0035
VPS33A	Vacuolar protein sorting-associated protein 33A	Q96AX1	0.2	0.0433
VPS35	Vacuolar protein sorting-associated protein 35	Q96QK1	0.3	0.0001
VRK1	Serine/threonine-protein kinase VRK1	Q99986	-0.5	0.0173
VTN	Somatomedin-B;Vitronectin;Vitronectin V10 subunit;Vitronectin V65 subunit	P04004	1.0	0.0008
VWF	von Willebrand antigen 2;von Willebrand factor	P04275	0.5	0.0039
WDR12	Ribosome biogenesis protein WDR12	Q9GZL7	-0.5	0.0021
WDR18	WD repeat-containing protein 18	Q9BV38	-0.4	0.0467

WDR3	WD repeat-containing protein 3	Q9UNX4	-0.4	0.0155
WDR36	WD repeat-containing protein 36	Q8NI36	-0.5	0.0400
YARS	Tyrosine--tRNA ligase, cytoplasmic;Tyrosine--tRNA ligase, cytoplasmic, N-terminally processed	P54577	-0.2	0.0326
YWHAH	14-3-3 protein eta	Q04917	-0.4	0.0323
ZMPSTE24	CAAX prenyl protease 1 homolog	O75844	-0.5	0.0010
ZNF598	Zinc finger protein 598	Q86UK7-2;Q86UK7-3;Q86UK7	-1.0	0.0088
ZYX	Zyxin	Q15942	-0.6	0.0423

Supplementary table 3. Differentially abundant transcripts between the two groups of patients

Gene name	log2FC (R vs S)	adjusted p-value
ABCA2	1.1	0.084
ABCD2	1.7	0.024
ALOX15B	1.9	0.036
ANGPTL4	1.6	0.100
APBB2	-1.3	0.088
AQP9	2.1	0.018
ARHGEF3	1.3	0.011
ARL6IP5	0.9	0.089
BCL11B	1.5	0.038
BHLHE40	0.9	0.067
BMP7	-1.8	0.053
BMP8B	1.9	0.038
BNC2	-1.3	0.075
BTBD9	0.8	0.004
BTD	0.8	0.089
C10orf35	1.6	0.089
C10orf47	1.5	0.070
C11orf21	1.6	0.070
C3	-1.4	0.060
CCL11	-1.9	0.050
CCL4	1.4	0.082
CCL5	1.3	0.088
CCNB1IP1	-1.0	0.075
CCR2	1.9	0.013
CD247	1.7	0.029
CD3E	1.5	0.054
CD3G	1.4	0.067
CD5	1.4	0.072
CD6	1.6	0.053
CD79B	-1.3	0.024
CD97	1.2	0.074
CDC42SE1	0.7	0.018
CDH23	1.5	0.099
CENPV	-2.0	0.018
CHI3L1	1.6	0.039
CHST1	-1.4	0.085
CIDEB	1.2	0.070
CLDN7	2.3	0.001
CLPTM1L	-0.7	0.070
COLEC12	-1.2	0.091
CP	1.7	0.083
CPM	1.5	0.055

CRAT	1.1	0.059
CRHBP	-1.5	0.079
CRTC3	1.1	0.025
CXCL13	1.6	0.019
CXCL5	1.7	0.088
DCBLD2	-1.1	0.075
EEF1G	-0.9	0.061
EMP3	1.0	0.050
EOMES	1.4	0.059
ERAP2	-1.2	0.060
FAM171B	-2.0	0.023
FAM186B	-1.8	0.023
FAM208B	-1.2	0.098
FAM78A	0.9	0.070
FBL	-0.8	0.070
FCGR2C	2.0	0.008
FGR	1.4	0.050
FKBP5	1.2	0.029
FMN1	1.3	0.067
FOXO3B	-1.3	0.100
FPR2	1.6	0.090
FURIN	1.1	0.059
G0S2	1.5	0.070
GBP5	1.7	0.056
GCSAM	-1.4	0.023
GIPR	1.4	0.059
GLB1L2	1.6	0.082
GLUL	1.5	0.059
GPBAR1	2.0	0.024
GRIP1	-1.8	0.058
GZMA	1.4	0.092
GZMH	1.7	0.047
GZMK	1.5	0.089
GZMM	1.5	0.067
HK3	1.9	0.028
HLA-C	1.4	0.089
HSPA12B	-1.5	0.067
HTRA1	-1.6	0.013
IDO1	1.7	0.061
IFNG	1.9	0.029
IL18RAP	1.9	0.038
IL21	1.7	0.089
IL2RB	1.5	0.038
IL32	1.4	0.024
IL4R	-1.1	0.059

IL6R	1.1	0.070
INPP4A	1.0	0.044
IRF2BPL	1.0	0.091
ITGA11	-1.7	0.067
ITGBL1	-1.6	0.091
ITK	1.7	0.018
ITPK1	0.8	0.060
KANK1	-2.1	0.000
KAT2B	1.0	0.079
KCNJ2	2.0	0.019
LAG3	1.5	0.070
LAMC3	1.8	0.055
LIMA1	-1.6	0.041
LMTK3	1.8	0.036
LOC100129269	-1.6	0.075
LOC100216546	-1.5	0.059
LOC100506888	-1.5	0.091
LOC100616530	-1.6	0.084
LTK	2.1	0.018
MARCH1	1.1	0.018
MARCH2	0.9	0.090
MARCO	2.2	0.018
MET	1.5	0.070
MGAT3	-1.2	0.095
MGC12916	1.5	0.061
MMP11	-1.3	0.093
MPND	1.0	0.044
MSX2	1.9	0.050
MT1F	1.8	0.039
MT1G	2.2	0.014
MT1H	1.6	0.080
MT1L	1.6	0.091
MT1M	2.5	0.004
MT1X	2.5	0.001
MT2A	2.4	0.002
MUC1	1.7	0.058
MYADML	-1.6	0.089
NACC2	1.3	0.059
NDRG1	1.4	0.053
NKG7	1.8	0.018
NLE1	-0.9	0.061
NRXN2	1.7	0.060
NSUN5	-0.8	0.067
NTM	-1.8	0.050
NUP85	-0.8	0.055

OBSCN	1.7	0.018
PALD1	-1.7	0.008
PCDHGB8P	-1.4	0.059
PDCD1	1.4	0.080
PDGFC	-1.4	0.075
PI15	1.9	0.039
PIAS3	0.7	0.088
PILRA	1.5	0.098
PLA2G16	1.1	0.088
PLCB2	1.1	0.045
PLCH2	1.6	0.059
PLTP	1.5	0.068
POTEH	1.6	0.094
PPM1L	-1.4	0.088
PPM1N	1.7	0.048
PREX1	1.1	0.067
PRND	-1.9	0.039
PTK6	1.5	0.070
QSOX2	-1.1	0.070
RAB27A	1.1	0.023
RCAN3	0.7	0.089
RFFL	0.9	0.070
RGN	-1.7	0.079
RIN3	1.2	0.050
RINT1	-1.0	0.060
RPL12	-1.0	0.029
RPL13	-1.1	0.092
RPL13A	-1.0	0.060
RPL18A	-0.9	0.091
RPL24	-0.8	0.059
RPL32	-0.9	0.067
RPL35A	-1.2	0.072
RPL36A	-1.0	0.088
RPL5	-1.0	0.029
RPS12	-0.9	0.089
RPS15A	-1.1	0.095
RPS18	-1.1	0.076
RPS6	-0.9	0.033
RPS8	-0.9	0.056
S100A4	1.4	0.020
S100A6	1.1	0.095
S100A8	1.7	0.066
SCARA5	1.6	0.089
SCARNA16	-1.3	0.067
SEMA4B	1.1	0.023

SERPINA1	1.8	0.039
SERPINB6	1.5	0.001
SH2B2	-1.5	0.011
SH2D1A	1.4	0.075
SIGIRR	1.5	0.014
SIGLEC14	2.2	0.004
SIRPG	1.6	0.045
SLC11A1	2.0	0.030
SLC25A35	1.0	0.073
SLC2A4RG	1.1	0.018
SLC43A1	-1.1	0.094
SLED1	1.5	0.060
SNHG4	-1.1	0.090
SNHG8	-1.1	0.076
SNORA1	-0.8	0.100
SNORA10	-1.3	0.060
SNORA14B	-1.1	0.029
SNORA21	-1.3	0.070
SNORA24	-1.6	0.060
SNORA27	-1.7	0.038
SNORA33	-1.5	0.059
SNORA53	-1.0	0.007
SNORA55	-1.3	0.089
SNORA56	-1.1	0.080
SNORA5C	-1.4	0.067
SNORA6	-1.3	0.089
SNORA62	-1.6	0.048
SNORA64	-1.5	0.089
SNORA65	-1.7	0.029
SNORA66	-1.4	0.076
SNORA67	-1.4	0.095
SNORA71D	-1.7	0.047
SNORA74A	-1.5	0.036
SNORA80B	-1.8	0.019
SNORA84	-1.0	0.089
SNORD15A	-1.9	0.018
SNORD17	-1.9	0.018
SNX10	1.5	0.070
SNX29P2	-1.6	0.024
SOBP	-1.3	0.059
SOD2	1.6	0.039
SORL1	-1.5	0.033
ST6GALNAC3	1.7	0.050
STEAP3	1.5	0.060
STOM	1.4	0.058

STX11	1.2	0.070
TBC1D9	1.2	0.067
TBX21	1.8	0.033
TCEB3C	-1.6	0.059
TCEB3CL	-1.5	0.089
TERT	-1.6	0.063
THEMIS2	1.3	0.067
THSD7A	-1.5	0.081
TMEM155	1.7	0.053
TMEM241	-1.5	0.023
TMEM98	-1.5	0.044
TMTC4	-1.3	0.060
TSPAN32	1.5	0.070
TSPAN6	-1.4	0.092
VSIG4	1.9	0.048
ZBED2	2.3	0.000
ZBTB16	1.7	0.060
ZC2HC1B	-2.3	0.009
ZC3HAV1L	-1.3	0.088
ZNF581	-1.1	0.019
ZNF608	-1.4	0.056
ZNF711	-2.0	0.018

Supplementary Table 4. Differentially expressed proteins not identified at the transcriptomic level.

**Gene names**	**Protein names**
Proteins overexpressed in chemorefractory patients and not identified at the transcriptomic level	
ECM29	Proteasome adapter and scaffold protein ECM29
FGA	Fibrinogen alpha chain
FGB	Fibrinogen beta chain
FGG	Fibrinogen gamma chain
MT-CO2	Cytochrome c oxidase subunit 2
PLG	Plasminogen
SERPIND1	Heparin cofactor 2
Proteins overexpressed in chemosensitive patients and not identified at the transcriptomic level	
AARSD1	Alanyl-tRNA editing protein Aarsd1
CLUH	Clustered mitochondria protein homolog
FAM107B	Protein FAM107B
GP1BB	Platelet glycoprotein Ib beta chain
HACD4	Very-long-chain (3R)-3-hydroxyacyl-CoA dehydratase 4
HLA-DQB1	HLA class II histocompatibility antigen, DQ beta 1 chain
NT5C3A	Cytosolic 5'-nucleotidase 3A
OLFM4	Olfactomedin-4
PBDC1	Protein PBDC1
RMDN1	Regulator of microtubule dynamics protein 1
RNASE3	Eosinophil cationic protein

Supplementary Table 5. Proteins with high expression at the proteomic level and low at the transcriptomic level, and the reverse, in chemorefractory and chemosensitive patients.

Gene names	Protein names
Proteins overexpressed in chemorefractory patients with a high fold-change at the proteomic level (log_2FC (R vs S) > 1) and low at the transcriptomic level (log_2FC (R vs S) < 1)	
ANXA6	Annexin A6
APOBEC3G	DNA dC->dU-editing enzyme APOBEC-3G
BLK	Tyrosine-protein kinase Blk
C4A	Complement C4-A
COL4A1	Collagen alpha-1(IV) chain
CSTA	Cystatin-A
DOK2	Docking protein 2
DSP	Desmoplakin
ECI1	Enoyl-CoA delta isomerase 1, mitochondrial
FBN1	Fibrillin-1
FCER1G	High affinity immunoglobulin epsilon receptor subunit gamma
GIMAP7	GTPase IMAP family member 7
GZMB	Granzyme B
IFI35	Interferon-induced 35 kDa protein
IVL	Involucrin
KLHL14	Kelch-like protein 14
LBP	Lipopolysaccharide-binding protein
LRRK2	Leucine-rich repeat serine/threonine-protein kinase 2
ME1	NADP-dependent malic enzyme
PLA2G4A	Cytosolic phospholipase A2
PTER	Phosphotriesterase-related protein
PYGL	Glycogen phosphorylase, liver form
SLC2A6	Solute carrier family 2, facilitated glucose transporter member 6
VTN	Vitronectin
Proteins overexpressed in chemorefractory patients with a low fold-change at the proteomic level (log_2FC (R vs S) < 1) and high at the transcriptomic level (log_2FC (R vs S) > 1)	
CD14	Monocyte differentiation antigen CD14
CD97	CD97 antigen
CP	Ceruloplasmin
CPPED1	Serine/threonine-protein phosphatase CPPED1
CTSB	Cathepsin B
DYSF	Dysferlin
GBP1	Guanylate-binding protein 1
GIMAP4	GTPase IMAP family member 4
GIMAP5	GTPase IMAP family member 5
GZMK	Granzyme K
MNDA	Myeloid cell nuclear differentiation antigen
MYO1F	Unconventional myosin-If
PREX1	Phosphatidylinositol 3,4,5-trisphosphate-dependent Rac exchanger 1 protein
S100A11	Protein S100-A11
S100A4	Protein S100-A4
SPN	Leukosialin

Proteins overexpressed in chemosensitive patients with a high fold-change at the proteomic level (log_2FC (R vs S) < -1) and low at the transcriptomic level (log_2FC (R vs S) > -1)	
ABI3BP	Target of Nesh-SH3
BGN	Biglycan
BPI	Bactericidal permeability-increasing protein
CAMP	Cathelicidin antimicrobial peptide
CLIP2	CAP-Gly domain-containing linker protein 2
COL12A1	Collagen alpha-1(XII) chain
FAM98A	Protein FAM98A
HLA-DOA	HLA class II histocompatibility antigen, DO alpha chain
HLA-DPB1	HLA class II histocompatibility antigen, DP beta 1 chain
ISLR	Immunoglobulin superfamily containing leucine-rich repeat protein 2
LXN	Latexin
MX2	Interferon-induced GTP-binding protein Mx2
OGN	Mimecan
OLFML1	Olfactomedin-like protein 1
PSMB6	Proteasome subunit beta type-6
RPRD1A	Regulation of nuclear pre-mRNA domain-containing protein 1A
SAMSN1	SAM domain-containing protein SAMSN-1
SDPR	Caveolae-associated protein 2
SLAMF1	Signaling lymphocytic activation molecule
SPTA1	Spectrin alpha chain, erythrocytic 1
TOMM22	Mitochondrial import receptor subunit TOM22 homolog
UTP20	Small subunit processome component 20 homolog
VCAN	Versican core protein
Proteins overexpressed in chemosensitive patients with a low fold-change at the proteomic level (log_2FC (R vs S) > -1) and high at the transcriptomic level (log_2FC (R vs S) < -1)	
ISG15	Ubiquitin-like protein ISG15
MYH11	Myosin-11
PRPF3	U4/U6 small nuclear ribonucleoprotein Prp3
RPS18	40S ribosomal protein S18
SLC25A13	Calcium-binding mitochondrial carrier protein Aralar2
